# The Organization of Controller Motifs Leading to Robust Plant Iron Homeostasis

**DOI:** 10.1371/journal.pone.0147120

**Published:** 2016-01-22

**Authors:** Oleg Agafonov, Christina Helen Selstø, Kristian Thorsen, Xiang Ming Xu, Tormod Drengstig, Peter Ruoff

**Affiliations:** 1 Centre for Organelle Research, University of Stavanger, Stavanger, Norway; 2 Department of Electrical Engineering and Computer Science, University of Stavanger, Stavanger, Norway; Louisiana State University Health Sciences Center, UNITED STATES

## Abstract

Iron is an essential element needed by all organisms for growth and development. Because iron becomes toxic at higher concentrations iron is under homeostatic control. Plants face also the problem that iron in the soil is tightly bound to oxygen and difficult to access. Plants have therefore developed special mechanisms for iron uptake and regulation. During the last years key components of plant iron regulation have been identified. How these components integrate and maintain robust iron homeostasis is presently not well understood. Here we use a computational approach to identify mechanisms for robust iron homeostasis in non-graminaceous plants. In comparison with experimental results certain control arrangements can be eliminated, among them that iron homeostasis is solely based on an iron-dependent degradation of the transporter IRT1. Recent IRT1 overexpression experiments suggested that IRT1-degradation is iron-independent. This suggestion appears to be misleading. We show that iron signaling pathways under IRT1 overexpression conditions become saturated, leading to a breakdown in iron regulation and to the observed iron-independent degradation of IRT1. A model, which complies with experimental data places the regulation of cytosolic iron at the transcript level of the transcription factor *FIT*. Including the experimental observation that FIT induces inhibition of IRT1 turnover we found a significant improvement in the system’s response time, suggesting a functional role for the FIT-mediated inhibition of IRT1 degradation. By combining iron uptake with storage and remobilization mechanisms a model is obtained which in a concerted manner integrates iron uptake, storage and remobilization. In agreement with experiments the model does not store iron during its high-affinity uptake. As an iron biofortification approach we discuss the possibility how iron can be accumulated even during high-affinity uptake.

## Introduction

**Fig 1:**
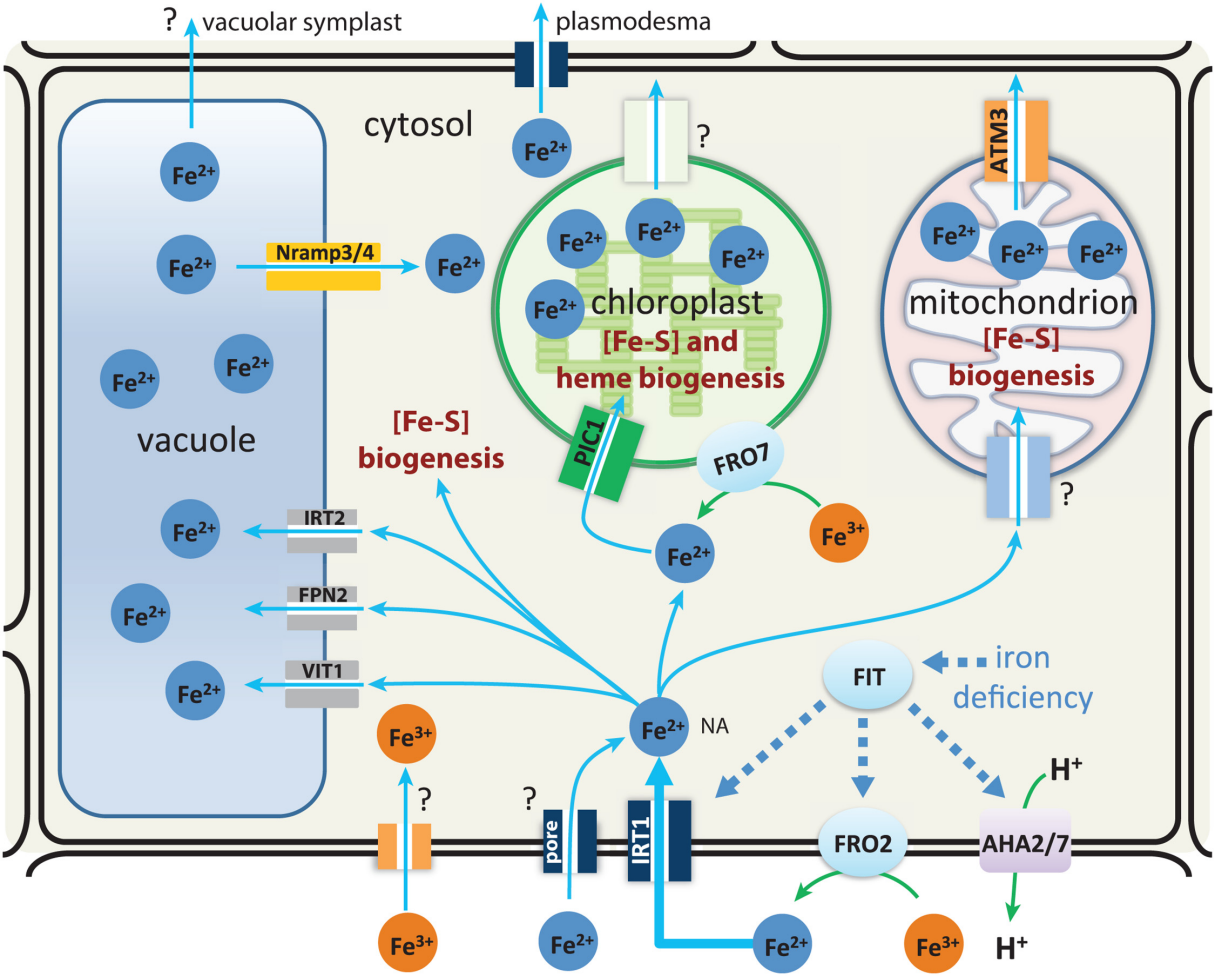
Regulatory components of iron homeostasis in non-graminaceous plant cells. In the presence of iron deficiency the transcription factor FIT activates the excretion of H^+^ by ATPases AHA2/7, which leads to an increased solubility of Fe(III). FIT also induces the reduction of solubilized Fe(III) to Fe(II) by the membrane-bound enzyme FRO2 and activates the high-affinity transporter IRT1 and the uptake of Fe(II). Iron is stored in different organelles with the vacuole as a major store. Several transporters which move iron into organelles and the vacuole have been identified (see main text). Iron transport to other parts of the plant occurs in complexed form with the water flow, i.e. via the cytosolic symplast connected by plasmodesmata and perhaps also by a transport route using a vacuolar symplast. Transporter candidates for iron remobilization from the store are Nramp3/4.

Iron is an essential element required by all organisms, but becomes toxic at higher levels. Iron is needed as a cofactor for many enzymes and proteins. To provide a sufficient level of available iron in the cytosol without leading to toxicity, iron is under homeostatic control. Plants have also the problem that iron in the soil under aerobic conditions is generally present as low-soluble iron(III)-oxide forms which require a high-affinity transport system for its uptake. To cope with these difficulties plants have developed two main strategies for iron-uptake, one termed strategy I for non-graminaceous plants (plants not belonging to the grass family) and the other termed strategy II for graminaceous plants [1]. During recent years considerable advances have been made to identify molecular components of the iron uptake and storage mechanisms [2–7]. In this work we focus on iron regulation of strategy I plants which includes the model plant *Arabidopsis thaliana*. Fig 1 gives an overview of the iron flow, storage, and regulatory components in these plants. IRT1 has been identified as the major transporter responsible for the high-affinity uptake of iron from the soil [8–10].

Prior to the uptake of iron(II) by IRT1, iron(III)’s solubility in the soil is increased by secreting H^+^ using H^+^-ATPases followed by the reduction of iron(III) to iron(II) by a membrane-bound ferric reductase oxidase (FRO2). The helix-loop-helix (bHLH) transcription factor FIT [11] has been found to be required for the iron deficiency response in Arabidopsis, where several iron regulated genes appear under the control of FIT [12]. FIT interacts with two other bHLH proteins, AtbHLH38 and AtbHLH39, where the transcription of *FRO2* and *IRT1* are regulated by FIT/AtbHLH38 and FIT/AtbHLH39 [13]. Once inside the cell, iron(II) is complexed and buffered by several organic compounds, among them Nicotianamine (NA), which is considered to stabilize predominantly Fe(II) but also Fe(III) [14] and appears to be an important transport form of iron in all plants [15–19].

By using *IRT1* knockout plants Vert et al. [10] observed that IRT1 is necessary for plant survival in the presence of low external iron levels, but that the absence of IRT1 plants can be counteracted by adding iron into the watering solution.

In accordance with results from metal-ion homeostasis in yeast [20], Connolly et al. [21] suggested a homeostasis-mediating mechanism based on the iron-dependent degradation of IRT1, where the amino acids K164 or K171 in IRT1 have been found to be necessary for IRT1 turnover [22]. This suggestion is supported by experiments [21] showing that the level of IRT1 (and its transcript) is high at low external iron concentrations, but declines once plants are transferred to sufficient iron conditions. In further agreement with an iron-dependent IRT1-degradation mechanism, 35S-*IRT1* plants which overexpress IRT1 in an Arabidopsis wild-type background have higher *IRT1* transcript levels, while protein levels were low and comparable to wild-type plants [22].

Barberon et al. [23] observed that overexpression of IRT1 in an *IRT1*-knockout background leads to elevated iron levels and toxicity. Furthermore, the IRT1 degradation rate under such conditions was found to be independent of the actual iron supply, in contrast to the earlier suggestion by Connolly et al. [21] of an iron-dependent degradation of IRT1. Despite the discrepancy whether IRT1 is subject to an iron-dependent degradation or not, there is a consensus that in roots mRNA and protein levels of *IRT1* and *FIT* are inversely correlated to the amount of external iron [10, 12, 21–23]. In other words, a sufficient supply of iron leads to low levels of *IRT1* and *FIT* transcripts and low levels of the corresponding proteins. Decreased supply levels of iron result in the up-regulation of both the *IRT1/FIT* transcripts and the corresponding proteins.

Chloroplasts require large amounts of iron due to photosynthesis, heme biosynthesis and Fe-S cluster synthesis. Besides chloroplasts, also mitochondria have a large demand for iron due to iron-containing respiratory enzymes. It is therefore expected that also these iron-requiring organelles have mechanisms to maintain iron homeostasis. Comparative studies of determined iron levels in roots and leaves performed with different wild-type and mutant plants show that roots have generally an iron content which is approximately one order of magnitude higher than in leaves (S1 Table). This indicates that in roots protective and homeostatic mechanisms need to be present in order to avoid iron toxicity while delivering iron to other parts of the plant.

The aim of this work is to rationalize, in agreement with experimental results, the organization of controller motifs, which lead to robust iron homeostasis in root cells during iron uptake, its assimilation and transport to other parts of the plant, as well as iron storage and its remobilization from the store. Before dealing with the aspects of iron regulation we give an overview of the concept of integral control, its importance for robust homeostasis and the kinetic implementations which lead to integral control in biochemical systems.

### Kinetic Requirements for Robust Homeostasis

The term *homeostasis* was introduced by Cannon in 1929 [24]. According to Cannon’s definition homeostasis maintains the steady states of compounds in an organism/cell at approximately constant and stable levels, where variations of these compounds may occur within certain but narrow limits [24–26]. Homeostasis is a concept which is closely related to the internal stability of organisms and cells [27]. Langley [26] provided an interesting compilation of key contributions which led to the development of the concept.

In control engineering it is well established that *integral control* (see e.g. [28]), as part of a negative feedback loop, will keep the level of a certain variable precisely at a given set-point even in the presence of environmental perturbations. Although the concept of integral control has extensively been used in industrial control processes since the last century its relevance with respect to the regulation of biochemical processes in organisms and cells was only relative recently pointed out [29] and studied in relation to several homeostatic processes [30–39].

We have been studying the kinetic requirements which lead to integral control in biochemical systems containing a negative feedback [33, 37, 38] including an extension of the homeostasis concept to oscillatory conditions [40]. To illustrate the effect of negative feedback regulation on a compound *A* consider the process:

$$\begin{array}{c} \overset{k_{1}}{\to }A\overset{k_{2}}{\to } \end{array}$$ (1)

where *k*_1_ and *k*_2_ are rate constants describing the inflow and outflow of *A* according to the rate equation:

$$\begin{array}{c} \dot{A}=k_{1}-k_{2}\cdot A \end{array}$$ (2)

Because the steady state level of *A* depends both on *k*_1_ and *k*_2_, i.e., *A*_*ss*_ = *k*_1_/*k*_2_, it is obvious that *A*_*ss*_ is not under homeostatic control.

**Fig 2:**
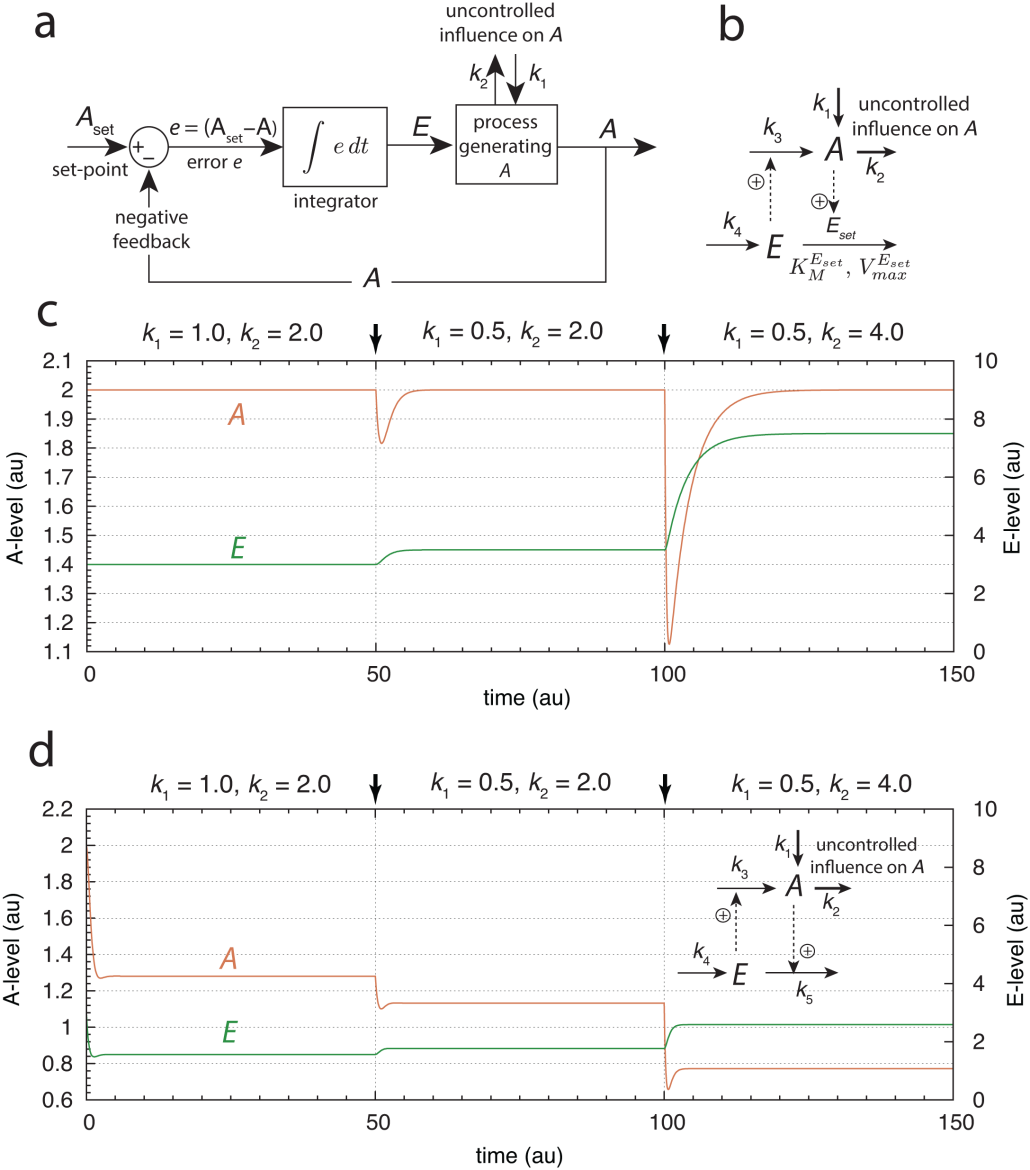
Negative Feedback Loops with and without Integral Control. (a) Flow diagram illustrating the concept of integral control. The regulated value of *A* is compared with its set-point *A*_*set*_ and the integral *E* of the error *e* between *A* and its set-point is calculated. *E* is fed into the process to compensate uncontrolled inflow or outflow to and from *A*. (b) Scheme of an inflow controller, where integral control is represented by removing *E* with enzyme *E*_*set*_, which is saturated with substrate E and reflected by a low $K_{M}^{E_{set}}$ value. (c) Illustration of robust homeostasis in *A* for different *k*_1_, *k*_2_ combinations with set-point $k_{4}/V_{max}^{E_{set}}$ Eq (6). The change in *k*_1_ and *k*_2_ occurs at *t* = 50.0 and *t* = 100.0 time units indicated by the arrows. Rate constants: *k*_3_ = 1.0, *k*_4_ = 2.0, $V_{max}^{E_{set}}=1.0$, and $K_{M}^{E_{set}}=1\times 10^{-4}$. Initial concentrations: *A*_0_ = 2.0, and *E*_0_ = 3.0. (d) Same negative feedback loop as in (b), but without integral control. The saturating kinetics of the *E*-removal is now replaced by a first-order process with respect to *E* with *k*_5_ = 1.0. The system is now not able to maintain robust homeostasis in *A*. Initial concentrations and the other rate constants are as in (c).

To keep the level of *A* at a certain set-point *A*_*set*_, integral control is invoked as part of a negative feedback controller loop. In integral control (Fig 2a) the difference (error *e*) between the actual value of *A* and its set-point *A*_*set*_, is calculated and integrated over time. The integrated error *E* is then used to compensate for perturbations in the concentration of A (for example when *k*_1_ or *k*_2_ are changed by environmental influences), which would drive the level of *A* away from its set-point. The controller loops can be divided into two classes, which we have termed *inflow* and *outflow* controllers [37]. An inflow controller provides a compensatory flux, which *adds* *A* from some other source to the system when uncontrolled perturbations decrease the level of *A*. In an outflow controller the situation is reversed, i.e., the compensatory flux *removes* *A* from the system by excreting *A* or moving it to a store [37]. Although a negative feedback loop is necessary to obtain robust homeostasis, negative feedback alone is not sufficient unless integral control is invoked. A justification of this statement is given in S1 Text. To illustrate the kinetic condition that leads to integral control and robust homeostasis, Fig 2b shows an inflow controller (motif 1, Ref. [37]), where integral control is implemented by an enzyme termed *E*_*set*_, which removes *E* under close to saturation conditions (low $K_{M}^{E_{set}}$ value). The name *E*_*set*_ reflects the enzyme’s importance for determining the set-point of A. The dashed arrows in Fig 2b with positive signs going from *A* to *E* indicate signaling events where *A* activates enzyme *E*_*set*_. In cybernetic terms the signaling from *A* to *E* is termed *measurement* and is part of the *A*-sensing mechanism of the controller. The dashed arrows from *E* to *A* indicate the signaling event where *E* activates the synthesis of *A*. In cybernetic terms this represents the *control input*, which is part of the compensatory mechanism and maintains homeostasis in *A*. For the sake of simplicity we assume here that the signaling events originating from *A* and *E* are proportional to the concentrations of *A* and *E*, respectively. The rate equations for *A* and *E* are written as:

$$\begin{array}{c} \dot{A}=k_{1}+k_{3}\cdot E-k_{2}\cdot A \end{array}$$ (3)

$$\begin{array}{c} \dot{E}=k_{4}-A\cdot \frac{V_{max}^{E_{set}}\cdot E}{K_{M}^{E_{set}}+E} \end{array}$$ (4)

Eq (4) determines the set-point for *A*. For $K_{M}^{E_{set}}⪡E$, the removal of *E* becomes zero-order with respect to *E* and Eq (4) simplifies to

$$\begin{array}{c} \dot{E}\approx k_{4}-V_{max}^{E_{set}}\cdot A=V_{max}^{E_{set}}(\frac{k_{4}}{V_{max}^{E_{set}}}-A)=V_{max}^{E_{set}}(A_{set}-A) \end{array}$$ (5)

We see that $\dot{E}$ is proportional to the error *e* = (*A*_*set*_ − *A*), and as required by integral control, the concentration of *E* is proportional to the integrated error (Fig 2a).

Under these conditions, the steady state concentration in *A* is given as

$$\begin{array}{c} A_{ss}=A_{set}=k_{4}/V_{max}^{E_{set}} \end{array}$$ (6)

and is independent of *k*_1_ and *k*_2_. Fig 2c illustrates the homeostatic behavior of the system when *k*_1_ and *k*_2_ values are varied.

The inset in Fig 2d shows the same negative feedback structure as in Fig 2b, but without the implementation of an integral controller. The zero-order removal of *E* in Fig 2b is now replaced by first-order kinetics:

$$\begin{array}{c} \dot{E}=k_{4}-k_{5}\cdot A\cdot E \end{array}$$ (7)

Because integral control with a defined set-point is lacking the steady state concentration of *A* (*A*_*ss*_) depends now on all four rate constants:

$$\begin{array}{c} A_{ss}=\frac{k_{1}+\sqrt{\frac{k_{1}^{2}\cdot k_{5}+4\cdot k_{2}\cdot k_{3}\cdot k_{4}}{k_{5}}}}{2\cdot k_{2}} \end{array}$$ (8)

Fig 2d shows the numerical results when the same changes in *k*_1_ and *k*_2_ are applied as in Fig 2c, but without integral control. In this case robust homeostasis cannot be maintained, although the steady state value of *A* for the negative feedback loop alone (Fig 2d) is higher than it would be without any negative feedback. Without a negative feedback the steady state would be *A*_*ss*_ = *k*_1_/*k*_2_, i.e., 0.5, 0.25 and 0.125 for the three different combinations of *k*_1_ and *k*_2_ in Fig 2d.

As we will show below, a negative feedback without integral control can still significantly affect another integral controller’s behavior by improving (decreasing) its response time while keeping the set-point unchanged.

## Materials and Methods

The experimental results on mRNA and protein levels we here will refer to have been reported in form of relative grayness levels of gel- and/or Western blots. Because experimentally determined cellular concentrations and associated rate parameters of compounds are still unknown, concentrations, rate constant and parameter values used in the models are kept in arbitrary units (au). However, to make the correspondence between modeling and experimental results as close as possible modeling results are reported, as experiments, in a blot-wise manner where gray levels reflect uncalibrated concentrations. According to the agreed convention in plant molecular biology, mRNAs are referred to in capitalized italic letters, while protein names are written capitalized and non-italic. Plants that had their *IRT1-gene* knocked-out are referred to with small italic letters, i.e. *irt1*. Computations were performed by using the Fortran subroutine LSODE [41] and compared with corresponding Matlab/Simulink calculations. Plots were generated with gnuplot (www.gnuplot.info) and Adobe Illustrator (adobe.com). To make notations simpler, concentrations of compounds are denoted by compound names without square brackets. To make the computational results available, Matlab files are provided as Supporting Information.

## Results and Discussion

### Negative Feedback with an Iron-dependent IRT1 degradation

We first investigated the suggestion by Connolly et al. [21] that IRT1 is degraded in an iron-dependent manner in comparison with the observation by Barberon et al. [23] that overexpression of IRT1 leads to an iron-independent degradation of IRT1.

**Fig 3:**
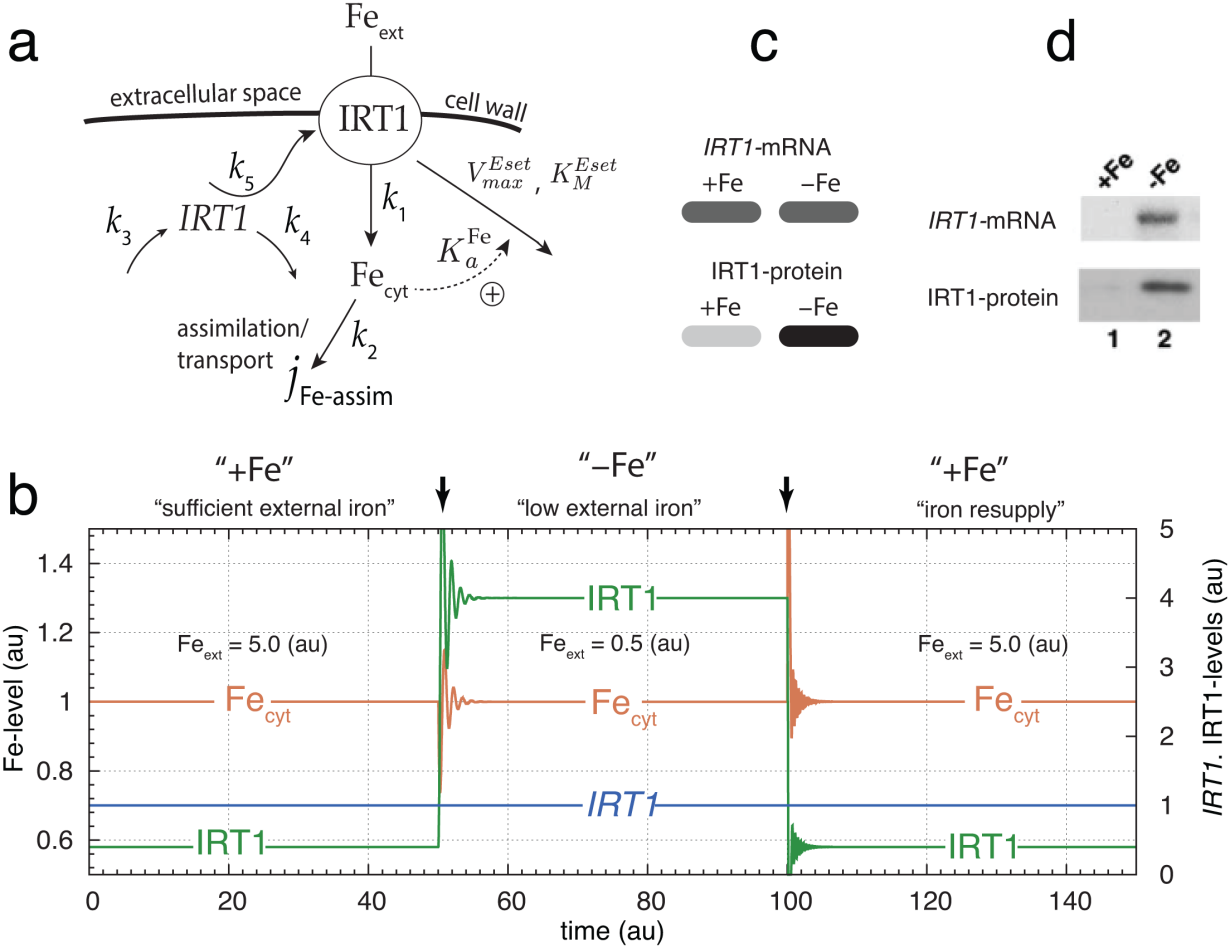
Regulatory loop of high affinity iron uptake and cytosolic iron homeostasis based on an iron-dependent IRT1 degradation. (a) Scheme of the control loop. Fe_ext_ and Fe_cyt_ denote external and cytosolic iron, respectively. *IRT1* and IRT1 denote mRNA and its protein, respectively. The extracellular iron concentration, Fe_ext_, is allowed to change to different but constant levels. (b) Homeostasis in cytosolic iron levels (Fe_cyt_) with respect to sufficient and low external iron conditions. The set-point for the level of cytosolic iron is given by Eq (12) and is arbitrarily set to ${\text{Fe}}_{\text{cyt}}^{\text{set}}=1.0$. In the first phase (time *t* = 0 to *t* = 50) Fe_ext_ = 5.0 and relative high. To keep iron at its homeostatic set-point during this phase the required concentration of IRT1 is relative low. In the second phase starting at *t* = 50 (arrow) Fe_ext_ is reduced to 0.5. Due to this reduction the IRT1 level is increased to keep the cytosolic iron concentration close at its set-point. In the third phase (*t* = 100 to *t* = 150) iron is resupplied and IRT1 levels decrease again. Other rate parameters remain unchanged during the three phases, i.e. *k*_1_ = 1.0, *k*_2_ = 2.0, *k*_3_ = 1.0 × 10^2^, *k*_4_ = 1.0 × 10^2^, *k*_5_ = 1.0 × 10^2^, $V_{max}^{Eset}=2.0\times 10^{2}$, $K_{M}^{Eset}=1.0\times 10^{-4}$, and $K_{a}^{\text{Fe}}=1.0$. Initial concentrations are Fe_cyt,0_ = 1.0, *IRT1*_0 = 1.0_, and IRT1_0 = 0.4_. (c) Representation of results in a ‘blot-like’ manner. +Fe and −Fe denote sufficient and low external ion conditions, respectively. For each component (*IRT1*, IRT1) the gray levels (0–100%) reflect the relative *IRT1*/IRT1 concentrations at +Fe and −Fe conditions. (d) Experimental data, slightly rearranged from Fig 6A in Ref. [21].

To understand these apparently opposing viewpoints we studied the model shown in Fig 3a, where iron homeostasis during iron uptake is based on an iron-dependent removal of the transporter protein IRT1. The inflow control structure of the model (the rate equations are given in S2 Text) is able to maintain homeostasis when the cellular demand for iron is relatively high.

For simplicity, the (high-affinity) uptake rate of iron by IRT1 is described as

$$\begin{array}{c} j_{\text{IRT1}}^{\text{Fe-uptake}}=k_{1}\cdot \text{IRT1}\cdot {\text{Fe}}_{\text{ext}} \end{array}$$ (9)

where $j_{\text{IRT1}}^{\text{Fe-uptake}}$ is proportional to the concentration of the transporter IRT1 and to the concentration of external iron, $\text{Fe}{}_{\text{ext}}$. Because the IRT1-based uptake of iron in general will show saturation kinetics, Eq (9) implies that iron uptake by IRT1 is far from saturation. By using isothermal titration calorimetry Grossoehme et al. [42] found that Fe^2+^ and other IRT1-transported metal-ions show a relative weak binding to IRT1 and that the description by Eq (9) appears approximately valid. To further simplify the model, the flux which maintains the cell’s need for iron and the transport flux of iron from the root to other parts of the plant are lumped together and described by the term *j*_Fe-assim_

$$\begin{array}{c} j_{\text{Fe-assim}}=k_{2}\cdot {\text{Fe}}_{\text{cyt}} \end{array}$$ (10)

The *IRT1*-mRNA (variable *IRT1*) is considered to be synthesized at a constant rate (*k*_3_) and degraded by a first-order process with respect to *IRT1*. The IRT1-transporter (variable IRT1) synthesis rate is proportional to the amount of *IRT1* transcript (variable *IRT1*). IRT1-protein is considered to be removed in an iron-dependent manner, where iron binds and thereby activates the IRT1-degrading enzyme *E*_*set*_. The fraction of activated enzyme removing IRT1 is given as $f_{a}^{\text{Fe}}$:

$$\begin{array}{c} f_{a}^{\text{Fe}}=\frac{{\text{Fe}}_{\text{cyt}}}{K_{a}^{\text{Fe}}+{\text{Fe}}_{\text{cyt}}} \end{array}$$ (11)

where $K_{a}^{\text{Fe}}$ is the dissociation constant between Fe_cyt_ and the nonactive form of the IRT1-degrading enzyme not having bound IRT1. The binding event between iron and the IRT1-removing enzyme can be seen as part of an iron sensing mechanism, where the amount of IRT1 reflects the concentration of cytosolic iron and may mediate this information to the regulation of other substances. Such a mechanism appears to be present to nitrate uptake in Arabidopsis where the nitrate transporters influences other uptake mechanisms [43, 44]. Due to the binding of iron to *E*_*set*_, $f_{a}^{\text{Fe}}$ has saturation properties. IRT1-degradation by the activated enzyme is described by a Michaelis-Menten type of reaction with a relative strong binding to its substrate, i.e. with a relative low $K_{M}^{E_{set}}$ value. Experiments indicate that IRT1 is degraded by the proteasome after ubiquinitation [23, 45]. The kinetics of the iron-induced IRT1 removal defines the set-point for cytosolic iron homeostasis. Setting both $\dot{\text{IRT1}}=0$ and $\dot{IRT1}=0$ together with $K_{M}^{E_{set}}⪡\text{IRT1}$, gives the following expression for the set-point (see S2 Text):

$$\begin{array}{c} {\text{Fe}}_{\text{cyt},set}=\frac{k_{3}\cdot k_{5}\cdot K_{a}^{\text{Fe}}}{k_{4}\cdot V_{max}^{E_{set}}-k_{3}\cdot k_{5}} \end{array}$$ (12)

The condition $K_{M}^{E_{set}}⪡\text{IRT1}$ represents an idealization used here for illustration, such that the controlled variable (here cytosolic iron) is kept at its set-point with high precision. However, it is presently not known at what degree of precision biochemical controllers usually operate. For controllers where $K_{M}^{E_{set}}$ values do not meet the condition $K_{M}^{E_{set}}⪡\text{IRT1}$ it has been shown that the value of $K_{M}^{E_{set}}$ is a direct measure of the controller’s accuracy [37].

Fig 3b shows the levels of Fe_cyt_, *IRT1*-mRNA and the IRT1 transporter for sufficient (“+Fe”) and low (“−Fe”) external iron conditions. The term ‘sufficent’ here means that the level of external iron is such that no significant up-regulation of IRT1 is necessary to meet the iron need of the cell/plant as expressed by the assimilation flux *j*_Fe-assim_ = *k*_2_⋅Fe_cyt_. In the case when the external iron concentration is low IRT1 needs up-regulation in order to meet the plant’s requirement for iron while keeping Fe_cyt_ close to its set-point.

Fig 3c shows the *IRT1* transcript and IRT1 protein levels in terms of a blot/gel-like view as would be obtained by Northern and Western blots, respectively. The relative concentrations in IRT1 at +Fe and −Fe conditions are expressed in terms of the gray percentage value, where the high IRT1 value of 4.0 (at “−Fe” condition) has been assigned a gray-level of 100% (black), while at the +Fe condition the gray-level has been reduced to 10% in accordance with the reduction of the IRT1-level to 0.4.

Ignoring for the moment the *IRT1* transcript data, the IRT1 protein dynamics of the controller are in good qualitative agreement with experimental results [10, 21, 23, 45], showing that at sufficient iron conditions, IRT1 levels are kept low but increase when iron becomes less available (Fig 3b and 3c). Fig 3d shows corresponding experimental data by Connolly et al. [21]. The up-regulation of *IRT1*-mRNA and IRT1-protein at iron-deficient conditions is clearly seen. When iron is resupplied, experiments and calculations show that IRT1-levels decrease again (see Fig 2 in [21] and Fig 3b).

### IRT1 Overexpression Leads to Saturation in Iron Signaling

**Fig 4:**
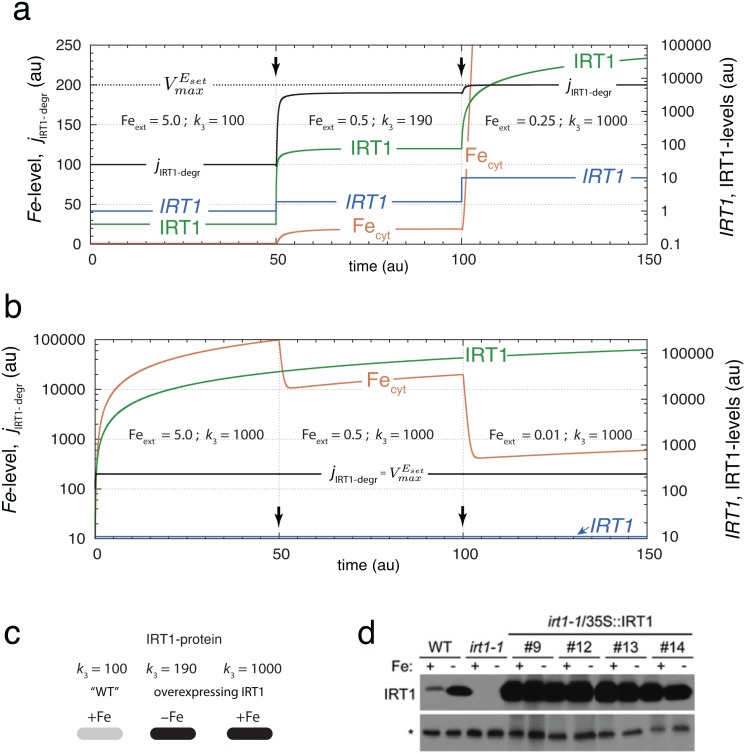
*IRT1* overexpression leads to an increased iron set-point, iron overload and to saturation in the iron-dependent degradation of IRT1. (a) Increase of *IRT1* synthesis rate *k*_3_ at different external iron levels. At times *t* = 50 and *t* = 100 (indicated by arrows) the values of Fe_ext_ and *k*_3_ are changed as indicated in the figure. As long as the IRT1 syntesis rate *j*_IRT1-synth_ is lower than its degradation rate (*t* = 0 to *t* = 100), the cytosolic iron concentration is under homeostatic control at its new set-point Eq (12). When *j*_IRT1-synth_ becomes larger than *j*_IRT1-degr_ iron levels rise and the IRT1 degradation rate *j*_IRT1-degr_ goes into saturation (*t* = 100 to *t* = 150). The negative feedback loop is broken and iron homeostasis lost. (b) Demonstration of iron-independent degradation of IRT1 when *j*_IRT1-synth_ > *j*_IRT1-degr_. The overexpression rate (*k*_3_) is kept constant at 1 × 10^3^ while external iron concentrations Fe_ext_ are changing. For each Fe_ext_ value (5.0, 0.5, and 0.01) the IRT1 degradation rate is at its maximum value $V_{max}^{E_{set}}$ and independent of the cytosolic iron concentration. Rate constants, except *k*_3_, are as in Fig 3c. (c) Calculated IRT1 expression levels shown as “dot-blots”. (d) Corresponding experimental results by Barberon et al. (Fig 1D in [23]).

When *IRT1* is over-expressed in plants with an *IRT1*-knockout background Barberon et al. [23] observed accumulation of IRT1, metal/iron overload, and oxidative stress. Under these conditions IRT1 degradation rates were found to be independent of the amount of supplied external iron, in contrast to the suggestion of an iron-dependent degradation of IRT1 considered in earlier work [21, 22]. We here show that under *IRT1* overexpression conditions the observation of iron overload and an iron-independent degradation of IRT1 can be rationalized by the homeostasis model in Fig 3a. In the model overexpression of *IRT1* is achieved by increasing *k*_3_. As *k*_3_ increases the set-point of cytosolic iron also increases see Eq (12) and leads to elevated levels of cytosolic iron, which can explain the observation [23] of iron overload. Fig 4a shows the model’s behavior when *k*_3_ is increased from 1.0 × 10^2^ to 1.9 × 10^2^ at *t* = 50. This increase in *k*_3_ leads to an increased set-point from 1.0 to 19.0. In addition, IRT1 levels are also increased in order to maintain homeostasis at the higher set-point. Although the homeostatic performance of the system is still functional at the new *k*_3_ value, the signaling pathway from iron to IRT1 degradation is reaching its capacity limit with a change of $f_{a}^{\text{Fe}}$ from 0.50 to 0.95. Accordingly, the rate of IRT1 degradation, *j*_IRT1-degr_, described by Eq (13) has moved close to its maximum level of $V_{max}^{E_{set}}$ (Fig 4a).

$$\begin{array}{c} j_{\text{IRT1-degr}}=\frac{{\text{Fe}}_{\text{cyt}}}{K_{a}^{\text{Fe}}+{\text{Fe}}_{\text{cyt}}}\cdot \frac{V_{max}^{E_{set}}\cdot \text{IRT1}}{K_{M}^{E_{set}}+\text{IRT1}} \end{array}$$ (13)

Once the synthesis rate of IRT1 ($j_{\text{IRT1-synth}}=k_{5}\cdot IRT1$) exceeds the capacity $V_{max}^{E_{set}}$ of the IRT1-degrading enzyme, IRT1-protein and cytosolic iron levels increase dramatically. This is shown in the third phase of Fig 4a when *k*_3_ = 1 × 10^3^. As a result of the large *j*_IRT1-synth_ flux, the signaling pathway from iron to IRT1 degradation becomes saturated, i.e., $f_{a}^{\text{Fe}}\to 1$, the negative feedback is no longer operational, and IRT1 levels rise. At this stage the IRT1 degradation rate becomes saturated and independent of the external iron concentration, as illustrated in Fig 4b. In Fig 4c the calculated overexpression results are replotted in form of dot blots. Fig 4d shows corresponding experimental results taken from Fig 1D by Barberon et al. [23]. In agreement with the modeling results for IRT1-protein (Fig 3c), the experimental results by Barberon et al. show the same wild-type (WT) regulation as previously observed by Connolly et al. [21] (Fig 3d) and others. However, for 35S-IRT1 overexpression conditions, the results by Barberon et al. [23] indicate a loss of IRT1-regulation by external iron (Fig 4d), while Fig 6B (lanes 1 and 2) by Connolly et al. [21] still shows such a regulation. This apparent disagreement between the 35S-IRT1 overexpression results can be rationalized by assuming that the IRT1 synthesis rate in the experiments by Connolly et al. rate is still below the capacity of the cell’s IRT1 degradation capacity, while for the IRT1 overexpression conditions by Barberon et al. the IRT1 synthesis rate has exceeded that capacity. The signaling event from cytosolic iron to the degradation machinery for IRT1 in Fig 3a can be interpreted to be part of the system’s iron sensing mechanism which breaks down. The breakdown/saturation of a still undiscovered iron sensing mechanism would be another alternative to interpret the iron-insensitivity of IRT1 degradation/inactivation at strong IRT1 overexpressing conditions. Thus, overexpression studies alone do not provide sufficient evidence to rule out an iron-dependent degradation/inactivation of IRT1 at normal operating conditions.

### Model including *IRT1*-mRNA and regulation by *FIT*

The model in Fig 3a did not include the regulation of *IRT1* transcript levels as indicated by the experiments shown in Fig 3d. In addition, the *FIT* gene was found to be essential for the high-affinity uptake of iron [10, 12, 21, 23, 46, 47]. The complex between AtbHLH38/AtbHLH39 and FIT has been found to activate *IRT1* and *FRO2* expression [13]. In addition, FIT was found to take part in the inhibiting of IRT1-protein degradation [12]. Model calculations shown below predict that the FIT-induced inhibition of IRT1 degradation has a role in improving (decreasing) the response time of the plant’s iron homeostatic system.

**Fig 5:**
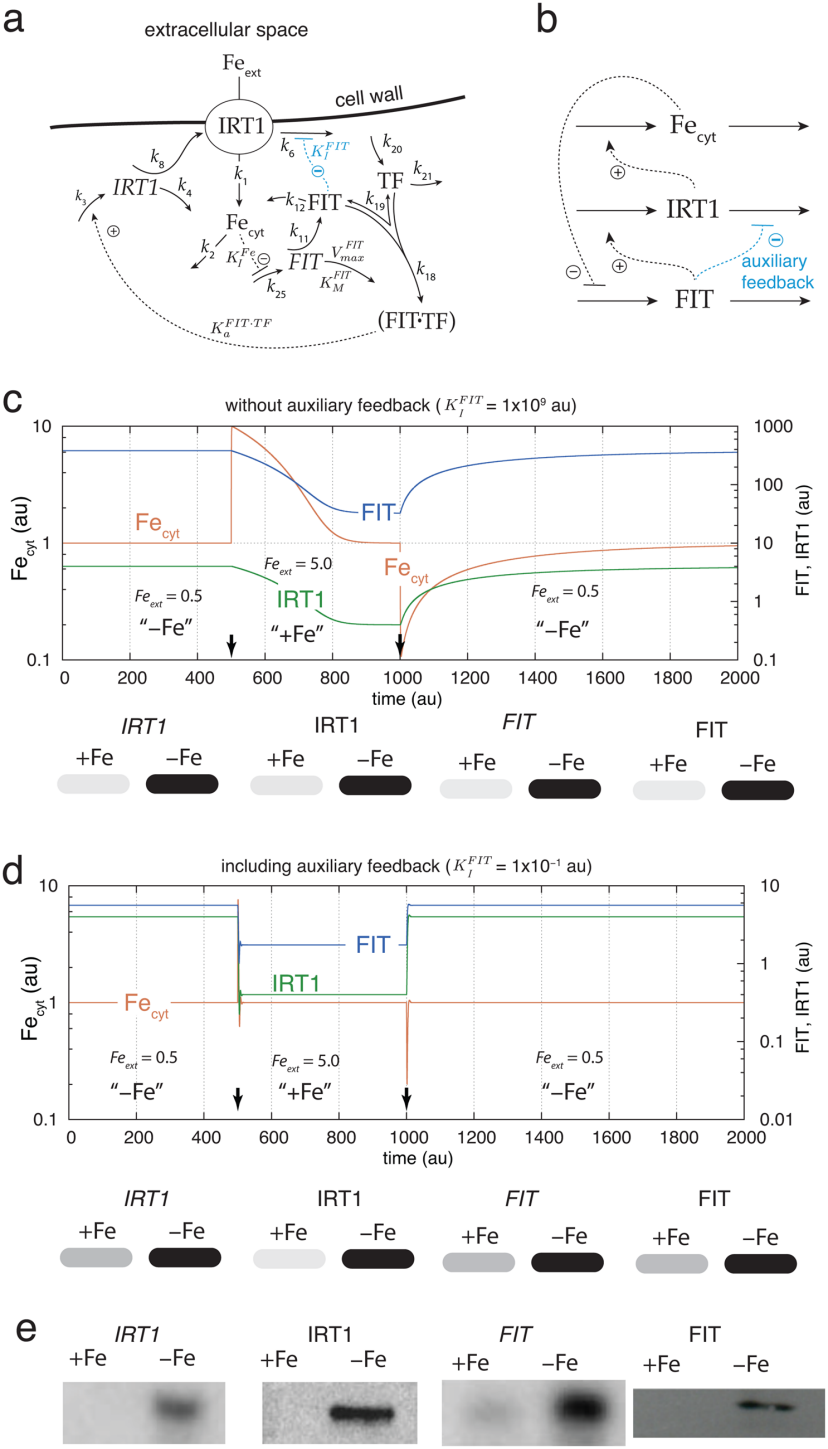
Model for iron uptake including *IRT1* and *FIT*. (a) Reaction scheme of the model. See S3 Text for rate equations. (b) Overview over the feedback structure of the model at the protein and cytosolic iron levels. The inhibition outlined in blue defines an additional auxiliary negative feedback which does not influence the set-point of cytosolic iron, but accelerates the adaptation kinetics of the controller (see (d)). (c) Regulation of *IRT1*- and *FIT*-mRNA and protein levels and response kinetics of the iron uptake system at low (-Fe) and high (+Fe) external iron concentrations and in the absence of the auxiliary feedback ($K_{I}^{\text{FIT}}=1\times 10^{9}$). The lower part of the panel shows the *IRT1*- and *FIT*-mRNA and protein levels in a blot-like representation, where high levels under -Fe conditions have a gray scale of 100%, while +Fe levels have a reduced gray scale in relation to their reduced numerical values. (d) Same as in (c), but now in the presence of the auxiliary feedback ($K_{I}^{\text{FIT}}=1\times 10^{-1}$). Note the improvement in the adaptation kinetics of the system. Rate constants for (c) and (d): *k*_1_ = 1.0, *k*_2_ = 2.0, *k*_3_ = 1 × 10^2^, *k*_4_ = 1.0, *k*_6_ = 4 × 10^2^, *k*_8_ = 1 × 10^2^, $K_{I}^{\text{FIT}}=1\times 10^{9}$, *k*_11_ = 1 × 10^3^, *k*_12_ = 1 × 10^3^, $K_{a}^{\text{FIT}\cdot \text{TF}}=1\times 10^{4}$, *k*_18_ = 1 × 10^2^, *k*_19_ = 1 × 10^1^, *k*_20_ = 1 × 10^4^, *k*_21_ = 2 × 10^4^, $K_{I}^{\text{Fe}}=1.0$, *k*_25_ = 4.0, $V_{max}^{FIT}=2.0$, $K_{M}^{FIT}=1\times 10^{-4}$. Initial concentrations for (c): Fe_cyt__0_ = 1.0, IRT1_0_ = 4.0, $IRT1_{0}=16.0$, FIT_0_ = 381.0, TF_0_ = 0.5, FIT⋅TF_0 = 1905.0_, $FIT_{0}=381.0$. Initial concentrations for (d): Fe_cyt__0_ = 1.0, IRT1_0_ = 4.0, $IRT1_{0}=0.3$, FIT_0_ = 5.6, TF_0_ = 0.5, FIT⋅TF_0 = 28.0_, $FIT_{0}=5.6$. (e) Experimental results of IRT1 and FIT mRNA and protein levels in wild-type Arabidopsis roots under iron sufficient (“+Fe”) and iron deficient (“-Fe”) conditions. The IRT1-protein and mRNA results as well as the *FIT*-mRNA blot are reproduced with permission from Fig 1 of Ref. [12]. The FIT-protein Western blot is reproduced with permission from Fig 4A of Ref. [47].

Fig 5a shows an extended model for the high-affinity uptake of iron and cytosolic iron homeostasis including *IRT1* and *FIT* transcript and protein levels. The rate equations of this model are given in S3 Text. The variable TF (Fig 5a) lumps together the transcription factors AtbHLH38 and AtbHLH39 which bind to FIT (FIT⋅TF) and activate the transcription of *IRT1* [13]. The activations of *FRO2* and AHA2/7 by FIT is not considered in the model. Many experiments confirm that the levels of *IRT1*- and *FIT*-mRNAs as well as IRT1 and FIT proteins are up- or down-regulated when external iron levels are decreased or increased, respectively. [10, 21, 23, 45–47]. The simplest way to rationalize the behaviors of these components is to place the homeostatic regulation point of cytosolic iron at the level of *FIT*-mRNA. An increase of *FIT*-mRNA levels when external and cytosolic iron are low (iron limiting conditions) will induce an increase in FIT-protein, and as a follow-up reaction, an increase in both *IRT1*-mRNA and IRT1 protein levels. Thus, separate regulatory loops for *IRT1*-mRNAs as well as FIT and IRT1-protein levels are in principle not necessary, although additional regulations are possible such as the identified FIT-induced inhibition of IRT1-degradation [12], which increase the performance of the homeostatic system. In the model, the regulation of *FIT*-mRNA is included in form of an iron-induced inhibition of *FIT*-mRNA synthesis. Alternatively, an iron-induced activation of *FIT*-mRNA degradation is possible, which shows practically the same up- and down-regulation characteristics of the components (data not shown). These are the only two possibilities of inflow controller motifs which match with the up- and down-regulation of the “E”-component [37] in the regulatory loop (here the “E”-component is the *FIT*-mRNA). There are presently no experimental indications favoring the inhibition of *FIT*-mRNA synthesis over an activation of its degradation or *vice versa*. For this model (Fig 5a) the set-point for cytosolic iron is determined by the rate equation for *FIT*:

$$\begin{array}{c} \dot{FIT}=\frac{k_{25}\cdot K_{I}^{\text{Fe}}}{K_{I}^{\text{Fe}}+{\text{Fe}}_{\text{cyt}}}-\frac{V_{max}^{FIT}\cdot FIT}{K_{M}^{FIT}+FIT} \end{array}$$ (14)

Setting $\dot{FIT}=0$ and using the condition/assumption that $K_{M}^{FIT}⪡FIT$ the following expression for the set-point of Fe_cyt_ is obtained

$$\begin{array}{c} {\text{Fe}}_{\text{cyt},set}^{FIT}=K_{I}^{\text{Fe}}(\frac{k_{25}}{V_{max}^{FIT}}-1) \end{array}$$ (15)

Fig 5b shows the outline of the feedback structures for the model in panel a. The inhibition of IRT1 degradation induced by FIT, outlined in blue, defines an additional experimentally identified negative feedback [12]. We incorporated this feedback, like that in Fig 2d, without an integral controller and with no influence upon the set-point value of Fe_cyt_ Eq (15).

Although having no influence on Fe_cyt_’s set-point, the feedback generated by the FIT-induced inhibition of IRT1 degradation, significantly improves the controller’s adaptation kinetics to the set-point (Fig 5c and 5d) and indicates a biological role for the FIT-induced inhibition of IRT1 degradation. We suggest to call this type of negative feedback loop for an ‘auxiliary feedback’.

Besides the inhibition of IRT1 degradation by FIT [12] several other additional negative feedback arrangements either in form of auxiliary feedbacks or containing an integral controller appear possible. An additional candidate for a helper feedback may be an iron-dependent degradation of FIT. Experiments have shown that the proteasomal degradation of FIT is necessary for the plant’s iron deficiency response [47, 48], but whether cytosolic iron regulates a proteasomal FIT degradation is presently not known. Also an iron-dependent degradation of IRT1 as previously suggested [21] can act as an additional auxiliary feedback.

The model results described in Fig 5c and 5d are in good agreement with corresponding experimental findings shown in Fig 5e where the results from different laboratories are shown, i.e., the respective up- and down-regulations of IRT1 and FIT transcript and protein levels at high and low external iron conditions.

### Iron Homeostasis Including Storage and Remobilization

At high external iron concentrations iron is taken up by an IRT1-independent mechanism [10], possibly due to a low-affinity uptake of iron by other metal-ion transporters [49–51].

Under high cytosolic iron concentrations the inflow controller in Fig 5a will automatically shut-down [37], while iron can still enter the cell and lead to high and potentially toxic iron levels [52]. To avoid the buildup of excess iron in the cytosol plants store and bind inflowing iron. One of the components is ferritin, a protein which is able to bind a large number of iron atoms. Ferritin is found in leaf chloroplasts [53] but also in mitochondria [54] probably reflecting the high abundance of iron in these organelles. Although ferritins are essential to protect the cell and its organelles from oxidative stress, ferritin is not considered to be a major iron pool for either seedling development or for the photosynthetic apparatus [52, 55]. Another molecule, Nicotianamine (NA), which is synthesized from three S-adenosylmethionine molecules, binds both Fe(II) and Fe(III). Although the binding constants are high for both oxidation states, Fe(II) is kinetically stabilized under aerobic conditions [14]. The NA-Fe(II) complex appears to be an important intracellular iron transport form for all plants and is a relative poor Fenton reagent [14, 16–18]. In the tomato mutant plant *chloronerva* where NA is nonfunctional due to a single base change [15], retarded growth of shoots and roots was observed, despite the fact that sufficient external iron was made available. In these plants precipitation of Fe(III)-phosphate occurred [56], providing an explanation why iron in *chloronerva* appeared less available.

Localization studies of NA, used as an indicator for the NA-Fe(II) complex and thereby for iron, revealed for different wild type plants that at low external iron concentrations most of the NA label appeared in the cytosol. For high iron loading conditions NA was found to be located in the vacuole [16]. This suggests that the vacuole acts as an iron store at sufficient high external iron concentrations, but that no vacuolar storage of iron occurs under iron-limiting conditions. In yeast, iron is stored as Fe(III), which during its remobilization from the vacuole is reduced by Fre6p to Fe(III) [57]. For plants, no corresponding metalloreductase has so far been found [58] indicating that the main storage form of iron in the vacuole appears to be complexed Fe(II).

A candidate for transporting iron into the vacuole is IRT2, a homolog to IRT1 which is co-regulated with FRO2 and IRT1 [59]. IRT2 is expressed in intracellular membranes. It has been suggested that IRT2 is part of an overflow mechanism [18], which sequesters iron into the vacuole or other non-characterized intracellular vesicles [60]. Other candidate transporters for moving cytosolic iron into the vacuole are VIT1 (during seed development) [61], FPN2 [62], a homolog of mammalian ferroportin and VTL [63]. In Arabidopsis, NRAMP3 and NRAMP4 take part in the iron remobilization from the vacuole into the cytosol during iron deficiency [18, 64, 65].

**Fig 6:**
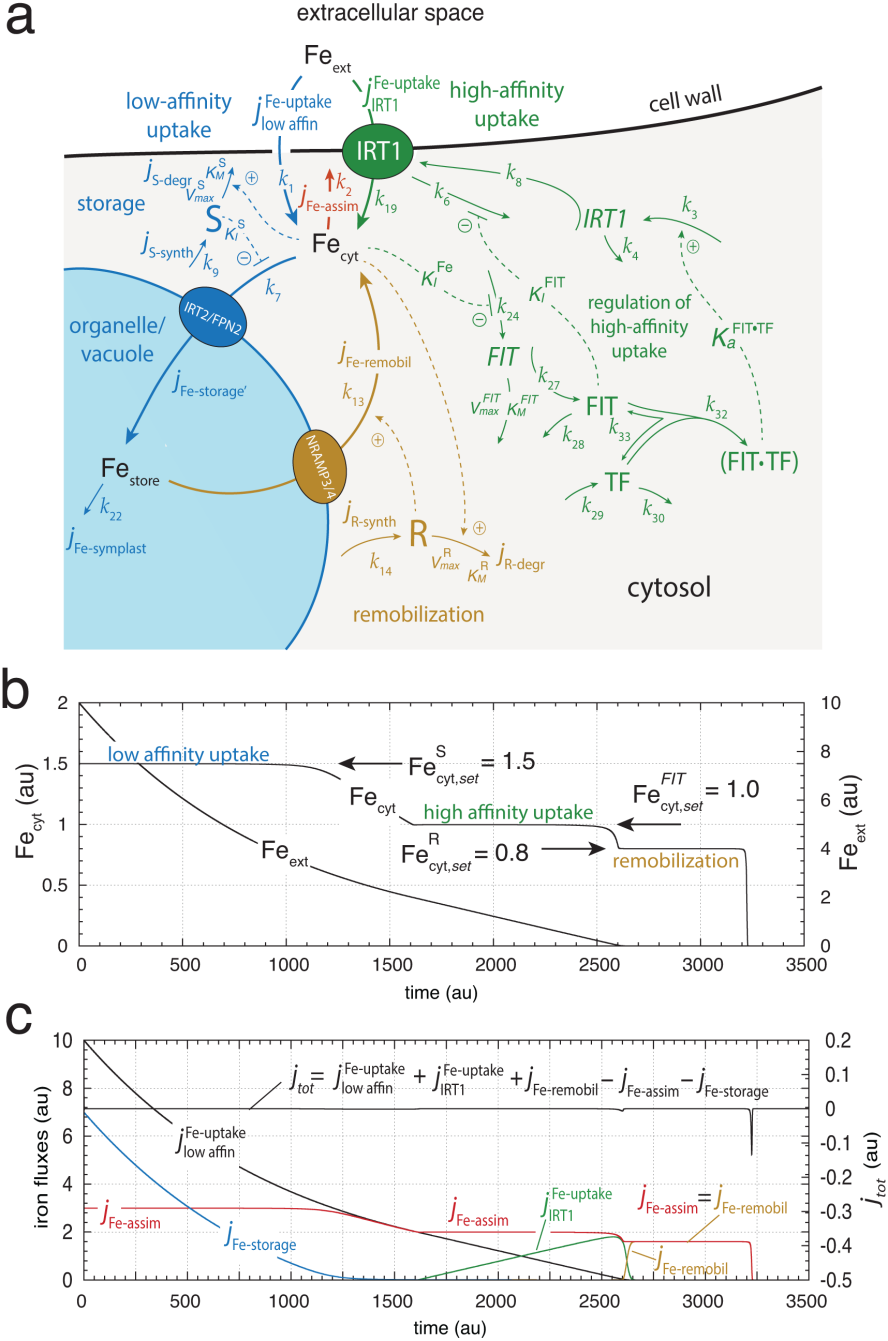
Model of plant iron homeostasis integrating uptake, storage, assimilation/transport and remobilization from the store. (a) The model combines a low-affinity iron uptake based on an iron-dependent derepression mechanism of inhibitor S [53], which leads to iron storage (outlined in blue), an R-based iron remobilization mechanism from the store (outlined in ochre), the FIT-based high-affinity iron uptake mechanism from Fig 5a (outlined in green) and a lumped expression for the iron assimilation and transport flux to other parts of the plant (outlined in red). Note the renumbering for some of the rate constants in comparison with Fig 5a. See S4 Text for rate equations. (b) Calculation showing cytosolic and external iron concentrations during the low- and high-affinity uptake of iron and iron remobilization from the vacuole. (c) Same calculation as in (b), but showing the different iron fluxes. Rate constants and initial concentrations for (b) and (c): *k*_1_ = 1.0, *k*_2_ = 2.0, *k*_3_ = 1 × 10^2^, *k*_4_ = 1.0, *k*_6_ = 4 × 10^2^, *k*_7_ = 1 × 10^2^, *k*_8_ = 1 × 10^2^, *k*_9_ = 15.0, $V_{max}^{\text{S}}=10.0$, $K_{M}^{\text{S}}=1\times 10^{-4}$, $K_{I}^{\text{S}}=0.1$, *k*_13_ = 0.5, *k*_14_ = 0.8, $V_{max}^{\text{R}}=1.0$, $K_{M}^{\text{R}}=1\times 10^{-6}$, *k*_19_ = 0.5, *k*_22_ = 5 × 10^−4^, $K_{I}^{\text{Fe}}=1.0$, *k*_24_ = 4.0, $V_{max}^{FIT}=2.0$, $K_{M}^{FIT}=1\times 10^{-4}$, *k*_27_ = 1 × 10^3^, *k*_28_ = 1 × 10^3^, *k*_29_ = 1 × 10^4^; *k*_30_ = 2 × 10^4^, *k*_32_ = 1 × 10^2^, *k*_33_ = 10.0, $K_{a}^{\text{FIT}\cdot \text{TF}}=1\times 10^{4}$, $K_{I}^{\text{FIT}}=0.01$. Fe_ext,0_ = 10.0, all other initial concentrations are zero.

Fig 6a shows a model integrating low- and high-affinity iron uptake with iron storage and iron remobilization. The S4 Text describes the rate equations. The change in cytosolic iron concentration can be expressed by the following fluxes

$$\begin{array}{c} \dot{{\text{Fe}}_{\text{cyt}}}=j_{\text{low}\;\text{affin}}^{\text{Fe-uptake}}+j_{\text{IRT1}}^{\text{Fe-uptake}}-j_{\text{Fe-assim}}-j_{\text{Fe-storage}}+j_{\text{Fe-remobil}} \end{array}$$ (16)

$j_{\text{low}\;\text{affin}}^{\text{Fe-uptake}}$ (outlined in blue) is the low affinity uptake rate of iron, which we for the sake of simplicity assumed to be proportional to the concentration of the external iron concentration Fe_ext_, i.e., not necessarily only diffusion-driven

$$\begin{array}{c} j_{\text{low}\;\text{affin}}^{\text{Fe-uptake}}=k_{1}\cdot {\text{Fe}}_{\text{ext}} \end{array}$$ (17)

$j_{\text{IRT1}}^{\text{Fe-uptake}}$ (outlined in green) is the high affinity uptake rate of iron

$$\begin{array}{c} j_{\text{IRT1}}^{\text{Fe-uptake}}=k_{19}\cdot \text{IRT1}\cdot {\text{Fe}}_{\text{ext}} \end{array}$$ (18)

where IRT1 is the concentration of IRT1-protein in the membrane.

*j*_Fe-assim_ (outlined in red) is the flux combining the assimilation of iron and its transport to other parts of the plant. This flux is described as Eq (10), i.e.

$$\begin{array}{c} j_{\text{Fe-assim}}=k_{2}\cdot {\text{Fe}}_{\text{cyt}} \end{array}$$ (19)

*j*_Fe-storage_ (outlined in blue) is the flux moving cytosolic iron into the store (the vacuole and other organelles). The activation of this flux is based on a mechanism which was described for the activation of ferritins in the presence of excess iron [53]. Under low iron conditions the transport of iron into the store is blocked by a still unknown inhibitor S. However, when iron inflow into the cytoplasm becomes high S is degraded by the proteosome in an iron-dependent manner and iron can enter the store [53]. The iron flux into the store is described by

$$\begin{array}{c} j_{\text{Fe-storage}}=k_{7}\cdot {\text{Fe}}_{\text{cyt}}\cdot (\frac{K_{I}^{\text{S}}}{K_{I}^{\text{S}}+\text{S}}) \end{array}$$ (20)

where $K_{I}^{\text{S}}$ is a inhibition constant by which S inhibits *j*_Fe-storage_. For the sake of simplicity the concentration of the transporters (IRT2/FPN2) moving iron into the store are considered to be constant, i.e., $k_{7}=k_{7}^{\prime }\cdot \left(\text{IRT2/FPN2}\right)=\text{constant}$, such that the flux of iron into the store is proportional to the concentration of Fe_cyt_. The inhibitor S may act at different levels, i.e. either directly inhibiting the transporter which moves iron into the store, or, like in the case of ferritin, acting at the transcriptional level [53].

The remobilization of iron from the store into the cytosol (ochre-colored) is formulated as

$$\begin{array}{c} j_{\text{Fe-remobil}}=k_{13}\cdot {\text{Fe}}_{\text{store}}\cdot \text{R} \end{array}$$ (21)

where R is the remobilization regulator and an inflow controller with respect to cytosolic iron. The rate equation of R is given as:

$$\begin{array}{c} \dot{\text{R}}=j_{\text{R-synth}}-j_{\text{R-degr}}=k_{14}-(\frac{V_{max}^{\text{R}}\cdot \text{R}}{K_{M}^{\text{R}}+\text{R}})\cdot {\text{Fe}}_{\text{cyt}} \end{array}$$ (22)

Also here the concentration of the transporters NRAMP3/4 is considered to be constant, i.e., $k_{13}=k_{13}^{\prime }\cdot \text{NRAMP3/4}=\text{constant}$. We are not aware of any identified feedback scheme with respect to iron remobilization from the vacuole. In this respect, the here suggested mechanism involving R is hypothetical. The set-point concentration of cytosolic iron with respect to inflow controller R is obtained by setting Eq (22) to zero and assuming that $K_{M}^{\text{R}}⪡\text{R}$. Solving for Fe_cyt_ gives

$$\begin{array}{c} {\text{Fe}}_{\text{cyt},set}^{\text{R}}=\frac{k_{14}}{V_{max}^{\text{R}}} \end{array}$$ (23)

The kinetics of the external iron source is described as

$$\begin{array}{c} \dot{{\text{Fe}}_{\text{ext}}}=-j_{\text{low}\;\text{affin}}^{\text{Fe-uptake}}-j_{\text{IRT1}}^{\text{Fe-uptake}} \end{array}$$ (24)

The rate equation for iron within the store is given by

$$\begin{array}{c} \dot{{\text{Fe}}_{\text{store}}}=j_{\text{Fe-storage}}-j_{\text{Fe-remobil}}-j_{\text{Fe-symplast}} \end{array}$$ (25)

Finally, we have the rate equation of the regulator S for iron storage:

$$\begin{array}{c} \dot{\text{S}}=j_{\text{S-synth}}-j_{\text{S-degr}} \end{array}$$ (26)

where *j*_S-synth_ is a constant (*k*_9_), while *j*_S-degr_ is activated by cytosolic iron and described by Michaelis-Menten kinetics

$$\begin{array}{c} j_{\text{S-degr}}={\text{Fe}}_{\text{cyt}}(\frac{V_{max}^{\text{S}}\cdot \text{S}}{K_{M}^{\text{S}}+\text{S}}) \end{array}$$ (27)

The set-point during storage is determined by setting $\dot{\text{S}}=0$ Eq (26), which leads to

$$\begin{array}{c} {\text{Fe}}_{\text{cyt},set}^{\text{S}}=\frac{j_{\text{S-synth}}}{V_{max}^{\text{S}}} \end{array}$$ (28)

To ensure that the combined controllers work flawlessly together, their set-points need to be in a certain hierarchical order as wind-up may occur otherwise [36, 37]. We have chosen the rate parameters such that ${\text{Fe}}_{\text{cyt},set}^{\text{S}}=1.5$, ${\text{Fe}}_{\text{cyt},set}^{FIT}=1.0$, and ${\text{Fe}}_{\text{cyt},set}^{\text{R}}=0.8$. The different set-point values also allow that the phases of storage, high-affinity uptake, and remobilization from the store can be easily identified (Fig 6b).

Fig 6b shows the concerted and integrative behavior of the combined controllers. The initial concentration of external iron, Fe_ext_, is 10.0 and relative high. At this condition the IRT1-based high-affinity uptake system is down-regulated and a still unknown uptake system moves iron into the cell [10]. To avoid a buildup of toxic iron within the cytosol, the excess of incoming iron (relative to the set-point of the S-controller) is moved by the S-controller into the vacuole/store. The negative feedback control loop for keeping homeostasis during storage (controller motif 6, [37]) is analogous to the motif identified for ferritin regulation ([53], see also above).

The flux into the store, *j*_Fe-storage_, decreases gradually with the decrease in the external iron concentration. At approximately 1250 time units (Fig 6b and 6c) there is not sufficient external ion available to maintain the homeostasis by the S-controller and the cytosolic iron concentration decreases (Fig 6b). As $j_{\text{low}\;\text{affin}}^{\text{Fe-uptake}}$ decreases the need for iron is satisfied by an increased IRT1-based uptake flux, $j_{\text{IRT1}}^{\text{Fe-uptake}}$, such that $j_{\text{low}\;\text{affin}}^{\text{Fe-uptake}}$ and $j_{\text{IRT1}}^{\text{Fe-uptake}}$ together compensate the assimilatory flux *j*_Fe-assim_ and maintain cytosolic iron homeostasis, i.e.,

$$\begin{array}{c} j_{\text{Fe-assim}}=j_{\text{low}\;\text{affin}}^{\text{Fe-uptake}}+j_{\text{IRT1}}^{\text{Fe-uptake}} \end{array}$$ (29)

The successive up-regulation of $j_{\text{IRT1}}^{\text{Fe-uptake}}$ continues until the external supply of iron is exhausted. In the model calculation this occurs at about 2600 time units (Fig 6b and 6c). In the final phase starting at about 2600 time units, the remobilization flux *j*_Fe-remobil_ from the store is activated and solely balances the assimilatory flux *j*_Fe-assim_.

**Fig 7:**
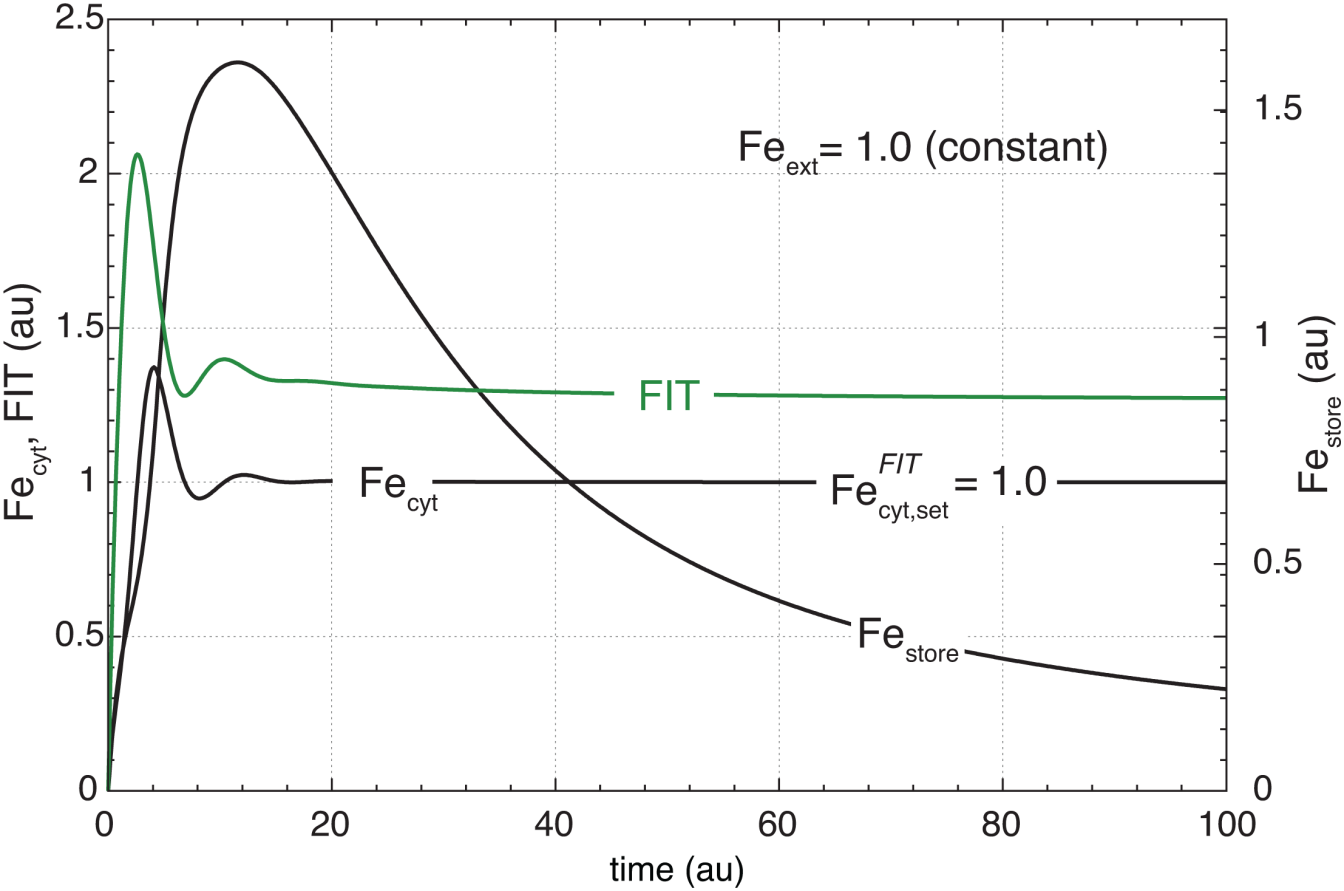
Iron-based derepression mechanism of iron storage is not active at low external iron concentrations. The figure shows that the iron-based derepression mechanism for iron storage (Fig 6a) cannot activate iron storage at low external iron concentrations when the IRT1 high-affinity uptake system is active. In this calculation the external iron concentration Fe_ext_ is kept constant at 1.0. Initial concentrations and rate constants for this calculation are as in Fig 6b and 6c, except for *k*_22_ (= 0.1) which accounts for a symplastic removal of iron out of the vacuole and leads to a decrease in Fe_store_. The FIT and IRT1-based high affinity uptake is able to keep the cytosolic iron at its set-point ${\text{Fe}}_{\text{cyt},set}^{FIT}=1.0$, while the S-based outflow controller (Fig 6a), responsible for iron storage, is inactive.

The arrangement of controller motifs in Fig 6a leads only to iron storage when a large amount of iron can enter the cell. In case the external iron concentration is low and iron becomes limiting, no significant storage of iron can occur. This is illustrated in Fig 7 showing the system’s response when the external iron concentration is kept at 1.0. While the FIT/IRT1-based controller balances the assimilatory flux *j*_Fe-assim_ no significant flux into the store occurs, and the store is emptied by the putative symplast iron transport [66, 67] which connects the vacuole to other parts of the plant (described by the flux *j*_Fe-symplast_ = *k*_22_⋅Fe_store_, Fig 6a). This strategy of vacuolar iron storage when there is only a surplus of external iron available fits well with experimental findings when NA-labelled plants are exposed to low and high external iron concentrations [16].

### Increasing Vacuolar Storage of Iron at Low External Iron Concentrations

**Fig 8:**
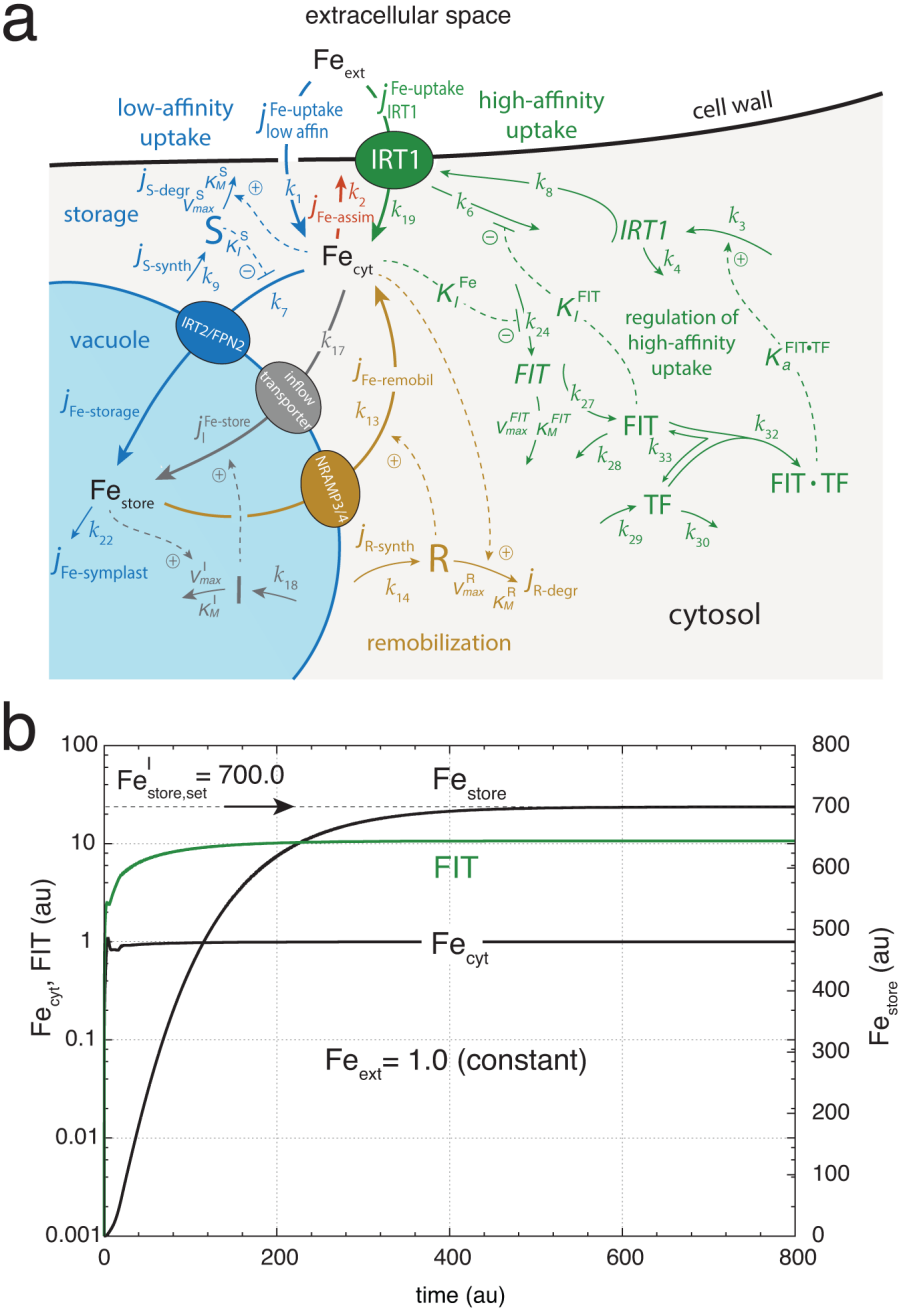
Iron storage during high-affinity uptake. (a) The model is an extension of that in Fig 6a containing in addition inflow controller molecule I inside the vacuole which activates a transporter located in the vacuolar membrane. Controller molecule I is subject to an iron-dependent degradation. The rate equation for I is: $\dot{\text{I}}=k_{18}-{\text{Fe}}_{\text{store}}\cdot V_{max}^{\text{I}}\cdot \text{I}/\left(K_{M}^{\text{I}}+\text{I}\right)$ (b) Calculation showing the increase of iron in the vacuole, Fe_store_, as a function of time. The iron set-point inside the vacuole is given by Eq (31) and set to 700.0. The flux of iron entering the vacuole due to controller I is given as: $j_{\text{I}}^{\text{Fe-store}}=k_{17}\cdot {\text{Fe}}_{\text{cyt}}\cdot \text{I}$. The additional parameter values are: *k*_17_ = 1 × 10^−3^, *k*_18_ = 700.0, $V_{max}^{\text{I}}=1.0$, $K_{M}^{\text{I}}=1\times 10^{-4}$. Other parameter values and initial concentrations as in Fig 6. The initial concentration of controller I is zero.

According to the World Health Organization iron deficiency is the most common and widespread nutritional disorder in the world [68]. As reviewed by Jeong and Guerinot [6] an understanding of iron homeostasis is not only important for getting better plant growth and increasing crop yields but also to improve human nutrition. Different approaches for iron biofortification in plants have been used, among them increasing the amount of ferritin and NA. Based on the properties of the here described controller motifs, we suggest a model-guided approach to increase the amount of stored iron in roots even when iron is taken up by the high-affinity system. The strategy is to place an inflow controller with respect to vacuolar iron within the vacuolar membrane while target the controller molecule into the vacuole (Fig 8a, outlined in gray). An inflow controller motif will try to maintain an iron homeostatic set-point in the vacuole defined by the negative feedback structure of the controller molecule I. Four controller motifs are in principle possible to achieve inflow control [37]. We here illustrate the approach using motif 1 [37] with an iron-induced degradation of controller I, while I is activating the inflow of iron into the vacuole (Fig 8a). The set-point of this controller is given by the ratio between I expression and its maximum degradation rate. An expression for the set-point is obtained by setting the rate equation for I to zero and solving for Fe_store_, i.e.,

$$\begin{array}{c} \dot{\text{I}}=k_{18}-{\text{Fe}}_{\text{store}}\cdot (\frac{V_{max}^{\text{I}}\cdot \text{I}}{K_{M}^{\text{I}}+\text{I}})=0 \end{array}$$ (30)

Assuming that $K_{M}^{\text{I}}⪡\text{I}$, the vacuolar iron set-point is given by

$$\begin{array}{c} {\text{Fe}}_{\text{store},set}^{\text{I}}=\frac{k_{18}}{V_{max}^{\text{I}}} \end{array}$$ (31)

Fig 8b illustrates the behavior of the system with a ${\text{Fe}}_{\text{store},set}^{\text{I}}=700$. Even at low iron conditions the system now accumulates iron in the vacuole, where ${\text{Fe}}_{\text{store},set}^{\text{I}}$ is an upper limit for the vacuolar iron concentration. At present this approach is purely theoretical, but transport routes to incorporate newly synthesized/overexpressed transporters such as FRP2 into the vacuolar membrane are known and can be used in a synthetic biology approach to generate iron accumulating plants. The usage of the vacuole to store increased amounts of iron has the advantage that increased iron concentration inside the vacuole are nontoxic to the plant, because FRP2-mediated transport of iron protects the plant from iron shock [69]. Although there has been made considerable progress in the understanding of plant iron regulation, our knowledge about the iron regulatory elements, their concentrations and kinetics are still fragmentary. We hope that the here suggested model-guided approach may stimulate further theoretical and experimental work leading to an increased understanding and better modification of iron regulation in higher plants.

### Hierarchical Arrangement of Inflow and Outflow Controllers

We have presented a model of plant iron homeostasis in root cells with emphasis on non-graminaceous plants/Arabidopsis, which integrates low- and high-affinity iron uptake, iron storage and remobilization as well iron assimilation and transport of iron to other parts of the plant. The model is based on a hierarchical arrangement of set-points when combining inflow and outflow controllers with respect to cytosolic iron. The outflow control arrangement (controller S) has the highest set-point (${\text{Fe}}_{\text{cyt},set}^{\text{S}}$) and moves iron from the cytoplasm to another part of the cell (store). An inflow controller associated with a high-affinity uptake system provides the next level of control with a set-point (${\text{Fe}}_{\text{cyt},set}^{FIT}$) below that of the outflow controller. This control level does not allow for storage, but balances the need of the cell for iron to maintain its functions while keeping the cytosolic iron concentration at a high enough level. The final level of control is that of remobilizing iron from the store back into the cytosol to balance the assimilatory flux and the need to maintain cellular function. The set-point of this control level (${\text{Fe}}_{\text{cyt},set}^{\text{R}}$) has the lowest value. This hierarchical arrangement of set-points

$$\begin{array}{c} {\text{Fe}}_{\text{cyt},set}^{\text{S}}>{\text{Fe}}_{\text{cyt},set}^{FIT}>{\text{Fe}}_{\text{cyt},set}^{\text{R}} \end{array}$$ (32)

allows for a concerted and cooperative manner of how the controllers are activated in response to external and internal iron supplies and requirements. Otherwise, if for example ${\text{Fe}}_{\text{cyt},set}^{FIT}<{\text{Fe}}_{\text{cyt},set}^{\text{R}}$ the store of iron would be always emptied *before* external iron is used by the high affinity uptake system. When ${\text{Fe}}_{\text{cyt},set}^{\text{S}}<{\text{Fe}}_{\text{cyt},set}^{FIT}$ the iron concentration will settle somewhere between both set-points, while both controllers are actively trying to move the cytosolic iron concentration to the level of their respective set-points. This behavior, when both S- and FIT-controllers are working “against each other” and leading to a constant upregulation of S, *FIT*-mRNA, and FIT-protein is referred to as “integral wind-up” [37]. To avoid windup, we anticipate that in all robustly homeostatic controlled systems a hierarchy of set-points is established as described by Eq (32) in order to maintain a concerted operation of the individual controllers.

### Sensor Mechanisms

Organisms need certain mechanisms to adapt to environmental changes. In this respect sensors and sensor mechanisms appear necessary to get information about the environment. The sequence of processes

$$\begin{array}{c} \text{sensing}\to \text{transduction}\to \text{reaction} \end{array}$$ (33)

has been considered as a general model how organisms and cells adapt to a changing environment [70]. In the literature [6] the anticipation of a not yet identified iron sensor upstream of *FIT* participating in iron homeostasis has been expressed. The regulatory structure in Fig 5b for the uptake of iron suggests an iron-sensing and signaling mechanism from Fe_cyt_ to *FIT*. As indicated above two possibilities exists for an inflow controller arrangement which matches the FIT and IRT1 dynamics upon external iron changes: either an inhibition of *FIT*-mRNA synthesis or an activation of its degradation. In addition to these sensing mechanisms as part of the regulatory negative feedback, there may be additional sensors that could, for example, be part of a feedforward control mechanism [71] to further optimize the system’s homeostatic response. In feedforward control environmental changes are measured/sensed and integrated into the negative feedback loop. Feedforward behavior is preset and appears to have developed due to an evolutionary process. Feedforward mechanisms are associated with anticipative behaviors of a system [72, 73] as found for example in circadian control [74, 75]. Plant iron has actually been found to be under a circadian regulation [53, 76]. Although not considered here, we have recently found that some of the controller motifs can be extended to work under oscillatory conditions [40], which will be a subject of further investigations.

## Supporting Information

S1 Table Overview of Determined Root and Leaf Iron Concentrations (*μ*g Fe per mg dry weight of tissue). (PDF)

S1 Text Robustness of Integral Control. (PDF)

S2 Text Dynamic model of Fig 3 and Derivation of Eq (12). (PDF)

S3 Text Dynamic model of Fig 5. (PDF)

S4 Text Dynamic model of Fig 6. (PDF)

S5 Text How to run the matlab files. (TXT)

S1 File matlab_fig2c.m file. (M)

S2 File ODEs_for_Figure_2c.m file. (M)

S3 File matlab_fig2d.m file. (M)

S4 File ODEs_for_Figure_2d.m file. (M)

S5 File matlab_fig3b.m file. (M)

S6 File ODEs_for_Figure_3b.m file. (M)

S7 File matlab_fig4a.m file. (M)

S8 File matlab_fig4b.m file. (M)

S9 File ODEs_for_Figure_4ab.m file. (M)

S10 File matlab_fig5c.m file. (M)

S11 File matlab_fig5d.m file. (M)

S12 File ODEs_for_Figure_5cd.m file. (M)

S13 File matlab_fig6.m file. (M)

S14 File ODEs_for_Figure_6bc.m file. (M)

S15 File matlab_fig7.m file. (M)

S16 File ODEs_for_Figure_7.m file. (M)

S17 File matlab_fig8b.m file. (M)

S18 File ODEs_for_Figure_8b.m file. (M)
