# Supplementary material for: The Organization of Controller Motifs Leading to Robust Plant Iron Homeostasis
Source: PLoS One. 2016 Jan 22;11(1):e0147120. doi: 10.1371/journal.pone.0147120 (PMC4723245; doi:10.1371/journal.pone.0147120)
Supplement: S1 Table — (PDF) [file pone.0147120.s001.pdf]

# The Organization of Controller Motifs Leading to Robust Plant Iron Homeostasis

Oleg Agafonov[1], Christina Helen Selstø[1], Kristian Thorsen[2], Xiang Ming Xu[1], Tormod Drengstig[2], Peter Ruoff[1,*]

**1 Centre for Organelle Research, University of Stavanger, Stavanger, Norway**
**2 Department of Electrical Engineering and Computer Science, University of Stavanger, Stavanger, Norway**
*** peter.ruoff@uis.no**

## Supporting Information

### S1 Table. Overview of Determined Root and Leaf Iron Concentrations ($\mu$g Fe per mg dry weight of tissue)

<table>
<tr><th>Plant species</th><th>strain</th><th>root</th><th>leaf</th><th>Reference</th></tr>
<tr><td rowspan="12">Arabidopsis</td><td>wild-type</td><td>3.2 $\mu$g/mg</td><td>0.15 $\mu$g/mg</td><td>[1]</td></tr>
<tr><td>wild-type</td><td>0.314 $\mu$g/mg</td><td>0.077 $\mu$g/mg</td><td>[2]</td></tr>
<tr><td>wild-type +Fe</td><td>3.2 $\mu$g/mg</td><td>0.16 $\mu$g/mg</td><td>[3]</td></tr>
<tr><td>wild-type −Fe</td><td>0.3 $\mu$g/mg</td><td>0.13 $\mu$g/mg</td><td>[3]</td></tr>
<tr><td>irt1-1 +Fe</td><td>3.5 $\mu$g/mg</td><td>0.17 $\mu$g/mg</td><td>[3]</td></tr>
<tr><td>irt1-1 −Fe</td><td>0.4 $\mu$g/mg</td><td>0.08 $\mu$g/mg</td><td>[3]</td></tr>
<tr><td>opt3-2</td><td>10 $\mu$g/mg</td><td>0.8 $\mu$g/mg</td><td>[1]</td></tr>
<tr><td>35S::IRT1 +Fe</td><td>3.0-4.4 $\mu$g/mg</td><td>0.21-0.37 $\mu$g/mg</td><td>[3]</td></tr>
<tr><td>35S::IRT1 −Fe</td><td>0.3-0.5 $\mu$g/mg</td><td>0.13-0.15 $\mu$g/mg</td><td>[3]</td></tr>
<tr><td>wild-type</td><td colspan="2">1.3 $\mu$g/mg</td><td>[4]</td></tr>
<tr><td>wild-type</td><td colspan="2">0.1 $\mu$g/mg</td><td>[5]</td></tr>
<tr><td>wild-type</td><td colspan="2">0.13 $\mu$g/mg</td><td>[6]</td></tr>
<tr><td rowspan="3">Tomato</td><td>wild-type −Fe</td><td>0.22 $\mu$g/mg</td><td>0.07 $\mu$g/mg</td><td>[7]</td></tr>
<tr><td>wild-type +Fe<br>10 $\mu$M Fe</td><td>2.38 $\mu$g/mg</td><td>0.14 $\mu$g/mg</td><td>[7]</td></tr>
<tr><td>wild-type +Fe<br>100 $\mu$M Fe</td><td>6.88 $\mu$g/mg</td><td>0.34 $\mu$g/mg</td><td>[7]</td></tr>
<tr><td rowspan="2">Rice</td><td>wild-type</td><td>18-30 $\mu$g/mg</td><td>0.6-0.85 $\mu$g/mg</td><td>[8]</td></tr>
<tr><td>wild-type</td><td>0.5-1.9 $\mu$g/mg)</td><td>0.27 $\mu$g/mg</td><td>[9]</td></tr>
</table>

## References

1. Mendoza-Cózatl DG, Xie Q, Akmakjian GZ, Jobe TO, Patel A, Stacey MG, et al. OPT3 is a component of the iron-signaling network between leaves and roots and misregulation of OPT3 leads to an over-accumulation of cadmium in seeds. Molecular plant. 2014;7(9):1455–1469.

2. Connolly EL, Fett JP, Guerinot ML. Expression of the IRT1 metal transporter is controlled by metals at the levels of transcript and protein accumulation. The Plant Cell. 2002;14(6):1347–1357.

3. Barberon M, Zelazny E, Robert S, Conéjéro G, Curie C, Friml J, et al. Monoubiquitin-dependent endocytosis of the iron-regulated transporter 1 (IRT1) transporter controls iron uptake in plants. PNAS. 2011;108(32):E450–E458.

4. Ward JT, Lahner B, Yakubova E, Salt DE, Raghothama KG. The effect of iron on the primary root elongation of Arabidopsis during phosphate deficiency. Plant Physiology. 2008;147(3):1181–1191.

5. Yuan Y, Wu H, Wang N, Li J, Zhao W, Du J, et al. FIT interacts with AtbHLH38 and AtbHLH39 in regulating iron uptake gene expression for iron homeostasis in Arabidopsis. Cell research. 2008;18(3):385–397.

6. Vert G, Grotz N, Dédaldéchamp F, Gaymard F, Guerinot ML, Briat JF, et al. IRT1, an Arabidopsis transporter essential for iron uptake from the soil and for plant growth. The Plant Cell. 2002;14(6):1223–1233.

7. Pich A, Manteuffel R, Hillmer S, Scholz G, Schmidt W. Fe homeostasis in plant cells: does nicotianamine play multiple roles in the regulation of cytoplasmic Fe concentration? Planta. 2001;213(6):967–976.

8. Silveira VCd, Oliveira APd, Sperotto RA, Espindola LS, Amaral L, Dias JF, et al. Influence of iron on mineral status of two rice (Oryza sativa L.) cultivars. Brazilian Journal of Plant Physiology. 2007;19(2):127–139.

9. Jia L, Wu Z, Hao X, Carrie C, Zheng L, Whelan J, et al. Identification of a novel mitochondrial protein, short postembryonic roots 1 (SPR1), involved in root development and iron homeostasis in Oryza sativa. New Phytologist. 2011;189(3):843–855.
