# Supplementary material for: The Organization of Controller Motifs Leading to Robust Plant Iron Homeostasis
Source: PLoS One. 2016 Jan 22;11(1):e0147120. doi: 10.1371/journal.pone.0147120 (PMC4723245; doi:10.1371/journal.pone.0147120)
Supplement: S1 Text — (PDF) [file pone.0147120.s002.pdf]

The Organization of Controller Motifs Leading to Robust Plant Iron Homeostasis

Oleg Agafonov[1], Christina Helen Selstø[1], Kristian Thorsen[2], Xiang Ming Xu[1], Tormod Drengstig[2], Peter Ruoff[1,*]

1 Centre for Organelle Research, University of Stavanger, Stavanger, Norway
2 Department of Electrical Engineering and Computer Science, University of Stavanger, Stavanger, Norway
*** peter.ruoff@uis.no**

Supporting Information

S1 Text. Robustness of Integral Control

Consider the flow-scheme in Fig. 2a. The robustness of the integral controller to keep A at A_{set} at steady state conditions can be shown as follows. The definition of integral control states that $E(t)$ is proportional to the integrated error $e(t) = (A_{set} - A(t))$

$$E(t) = G_i \int_0^t (A_{set} - A(\tau))\, d\tau \tag{1}$$

where A_{set} is the set-point and a constant.

At steady state conditions E and A do not change. From the condition $\dot{E} = 0$, we get

$$\dot{E(t)} = G_i(A_{set} - A_{ss}(t)) = 0 \qquad \Rightarrow \qquad A_{ss}(t) = A_{set} = \text{constant} \tag{2}$$

This shows that at steady state conditions and when integral control is operative A is kept robustly at A_{set}.
