# Supplementary material for: The Organization of Controller Motifs Leading to Robust Plant Iron Homeostasis
Source: PLoS One. 2016 Jan 22;11(1):e0147120. doi: 10.1371/journal.pone.0147120 (PMC4723245; doi:10.1371/journal.pone.0147120)
Supplement: S2 Text — (PDF) [file pone.0147120.s003.pdf]

The Organization of Controller Motifs Leading to Robust Plant Iron Homeostasis

Oleg Agafonov[1], Christina Helen Selstø[1], Kristian Thorsen[2], Xiang Ming Xu[1], Tormod Drengstig[2], Peter Ruoff[1,*]

1 Centre for Organelle Research, University of Stavanger, Stavanger, Norway
2 Department of Electrical Engineering and Computer Science, University of Stavanger, Stavanger, Norway
*** peter.ruoff@uis.no**

Supporting Information

S2 Text. Dynamic model of Fig. 3 and Derivation of Eq. 12

The model in Fig. 3 is described by the rate equations:

$$\dot{IRT1} = k_3 - k_4 \cdot IRT1 \tag{1}$$

$$\dot{\mathrm{IRT1}} = k_5 \cdot IRT1 - \left(\frac{\mathrm{Fe_{cyt}}}{K_a^{\mathrm{Fe}} + \mathrm{Fe_{cyt}}}\right) \cdot \left(\frac{V_{max}^{E_{set}} \cdot \mathrm{IRT1}}{K_M^{E_{set}} + \mathrm{IRT1}}\right) \tag{2}$$

$$\dot{\mathrm{Fe}}_{\mathrm{cyt}} = k_1 \cdot \mathrm{IRT1} \cdot \mathrm{Fe_{ext}} - k_2 \cdot \mathrm{Fe_{cyt}} \tag{3}$$

At steady state conditions $\dot{IRT1}$, $\dot{\mathrm{IRT1}}$, and $\dot{\mathrm{Fe}}_{\mathrm{cyt}}$ are zero, which give the steady state values $IRT1_{ss}$, $\mathrm{IRT1}_{ss}$, and $\mathrm{Fe}_{\mathrm{cyt},ss}$. The expression for $\mathrm{Fe}_{\mathrm{cyt},ss}$ also defines the set-point for cytosolic iron, $\mathrm{Fe}_{\mathrm{cyt},set}$.

Setting Eq. 37 to zero gives:

$$k_5 \cdot IRT1_{ss} = \left(\frac{\mathrm{Fe}_{\mathrm{cyt},ss}}{K_a^{\mathrm{Fe}} + \mathrm{Fe}_{\mathrm{cyt},ss}}\right) \cdot \left(\frac{V_{max}^{E_{set}} \cdot \mathrm{IRT1}_{ss}}{K_M^{E_{set}} + \mathrm{IRT1}_{ss}}\right) \tag{4}$$

When $K_M^{E_{set}} \ll \mathrm{IRT1}_{ss}$, Eq. 4 reduces to:

$$k_5 \cdot IRT1_{ss} = \left(\frac{\mathrm{Fe}_{\mathrm{cyt},ss}}{K_a^{\mathrm{Fe}} + \mathrm{Fe}_{\mathrm{cyt},ss}}\right) \cdot V_{max}^{E_{set}} \tag{5}$$

Inserting the $IRT1_{ss}$ value (k_3/k_4) into Eq. 5 and solving for $\mathrm{Fe}_{\mathrm{cyt},ss}$, gives Eq. 12, i.e.

$$\mathrm{Fe}_{\mathrm{cyt},ss} = \mathrm{Fe}_{\mathrm{cyt},set} = \frac{k_3 \cdot k_5 \cdot K_a^{\mathrm{Fe}}}{k_4 \cdot V_{max}^{E_{set}} - k_3 \cdot k_5} \tag{6}$$

Note that Eq. 6 (Eq. 12) is only valid as long as $\mathrm{Fe}_{\mathrm{cyt},ss}$ is finite. This is assured by the condition $V_{max}^{E_{set}} > \frac{k_3 \cdot k_5}{k_4}$. In kinetic terms this means that the steady state flux of IRT1 synthesis given by $j_{synth}^{\mathrm{IRT1}} = k_5 \cdot IRT1_{ss} = \frac{k_3 \cdot k_5}{k_4}$ should not exceed the IRT1 maximum degradation rate $V_{max}^{E_{set}}$. Once $j_{synth}^{\mathrm{IRT1}}$ exceeds $V_{max}^{E_{set}}$, i.e. $V_{max}^{E_{set}} \leq \frac{k_3 \cdot k_5}{k_4}$, the system becomes unstable, $\mathrm{Fe}_{\mathrm{cyt},ss}$ continuously increases and Eq. 41 is no longer valid.
