# Supplementary material for: The Organization of Controller Motifs Leading to Robust Plant Iron Homeostasis
Source: PLoS One. 2016 Jan 22;11(1):e0147120. doi: 10.1371/journal.pone.0147120 (PMC4723245; doi:10.1371/journal.pone.0147120)
Supplement: S3 Text — (PDF) [file pone.0147120.s004.pdf]

The Organization of Controller Motifs Leading to Robust Plant Iron Homeostasis

Oleg Agafonov[1], Christina Helen Selstø[1], Kristian Thorsen[2], Xiang Ming Xu[1], Tormod Drengstig[2], Peter Ruoff[1,*]

1 Centre for Organelle Research, University of Stavanger, Stavanger, Norway
2 Department of Electrical Engineering and Computer Science, University of Stavanger, Stavanger, Norway
*** peter.ruoff@uis.no**

Supporting Information

S3 Text. Dynamic model of Fig. 5

The model of Fig. 5 is:

$$\dot{\mathrm{Fe}_{\mathrm{cyt}}} = k_1 \cdot \mathrm{Fe}_{\mathrm{ext}} \cdot \mathrm{IRT1} - k_2 \cdot \mathrm{Fe}_{\mathrm{cyt}} \tag{1}$$

$$\dot{\mathrm{IRT1}} = k_8 \cdot IRT1 - k_6 \cdot \mathrm{IRT1} \cdot \left(\frac{K_I^{\mathrm{FIT}}}{K_I^{\mathrm{FIT}} + \mathrm{FIT}} \right) \tag{2}$$

$$\dot{IRT1} = k_3 \cdot \left(\frac{(\mathrm{FIT} \cdot \mathrm{TF})}{K_a^{(\mathrm{FIT} \cdot \mathrm{TF})} + (\mathrm{FIT} \cdot \mathrm{TF})} \right) - k_4 \cdot IRT1 \tag{3}$$

$$\dot{\mathrm{FIT}} = k_{11} \cdot FIT - k_{12} \cdot \mathrm{FIT} - k_{18} \cdot \mathrm{FIT} \cdot \mathrm{TF} + k_{19} \cdot (\mathrm{FIT} \cdot \mathrm{TF}) \tag{4}$$

$$\dot{\mathrm{TF}} = k_{20} - k_{21} \cdot \mathrm{TF} + k_{19} \cdot (\mathrm{FIT} \cdot \mathrm{TF}) - k_{18} \cdot \mathrm{FIT} \cdot \mathrm{TF} \tag{5}$$

$$(\mathrm{FIT} \dot{\cdot} \mathrm{TF}) = k_{18} \cdot \mathrm{FIT} \cdot \mathrm{TF} - k_{19} \cdot (\mathrm{FIT} \cdot \mathrm{TF}) \tag{6}$$

$$\dot{FIT} = k_{25} \cdot \left(\frac{K_I^{\mathrm{Fe}}}{K_I^{\mathrm{Fe}} + \mathrm{Fe}_{\mathrm{cyt}}} \right) - \frac{V_{max}^{FIT} \cdot FIT}{K_M^{FIT} + FIT} \tag{7}$$
