# Supplementary material for: The Organization of Controller Motifs Leading to Robust Plant Iron Homeostasis
Source: PLoS One. 2016 Jan 22;11(1):e0147120. doi: 10.1371/journal.pone.0147120 (PMC4723245; doi:10.1371/journal.pone.0147120)
Supplement: S4 Text — (PDF) [file pone.0147120.s005.pdf]

The Organization of Controller Motifs Leading to Robust Plant Iron Homeostasis

Oleg Agafonov[1], Christina Helen Selstø[1], Kristian Thorsen[2], Xiang Ming Xu[1], Tormod Drengstig[2], Peter Ruoff[1,*]

1 Centre for Organelle Research, University of Stavanger, Stavanger, Norway
2 Department of Electrical Engineering and Computer Science, University of Stavanger, Stavanger, Norway
*** peter.ruoff@uis.no**

Supporting Information

S4 Text. Dynamic model of Fig. 6

The model of Fig. 6 is:

$$\dot{\mathrm{Fe}}_{\mathrm{cyt}}=k_1\cdot \mathrm{Fe}_{\mathrm{ext}}+k_{19}\cdot \mathrm{IRT1}\cdot \mathrm{Fe}_{\mathrm{ext}}-k_2\cdot \mathrm{Fe}_{\mathrm{cyt}}-k_7\cdot \mathrm{Fe}_{\mathrm{cyt}}\cdot\left(\frac{K_I^{\mathrm{S}}}{K_I^{\mathrm{S}}+\mathrm{S}}\right)+k_{13}\cdot \mathrm{R}\cdot \mathrm{Fe}_{\mathrm{store}} \quad (1)$$

$$\dot{\mathrm{S}}=k_9-\mathrm{Fe}_{\mathrm{cyt}}\cdot\left(\frac{V_{max}^{\mathrm{S}}\cdot \mathrm{S}}{K_M^{\mathrm{S}}+\mathrm{S}}\right) \quad (2)$$

$$\dot{\mathrm{Fe}}_{\mathrm{store}}=k_7\cdot\left(\frac{K_I^{\mathrm{S}}}{K_I^{\mathrm{S}}+\mathrm{S}}\right)\cdot \mathrm{Fe}_{\mathrm{cyt}}-k_{13}\cdot \mathrm{R}\cdot \mathrm{Fe}_{\mathrm{store}}-k_{22}\cdot \mathrm{Fe}_{\mathrm{store}} \quad (3)$$

$$\dot{\mathrm{R}}=k_{14}-\mathrm{Fe}_{\mathrm{cyt}}\cdot\left(\frac{V_{max}^{\mathrm{R}}\cdot \mathrm{R}}{K_M^{\mathrm{R}}+\mathrm{R}}\right) \quad (4)$$

$$\dot{IRT1}=k_3\cdot\left(\frac{(\mathrm{FIT}\cdot \mathrm{TF})}{K_a^{(\mathrm{FIT}\cdot \mathrm{TF})}+(\mathrm{FIT}\cdot \mathrm{TF})}\right)-k_4\cdot IRT1 \quad (5)$$

$$\dot{\mathrm{IRT1}}=k_8\cdot IRT1-k_6\cdot\left(\frac{K_I^{\mathrm{FIT}}}{K_I^{\mathrm{FIT}}+\mathrm{FIT}}\right)\cdot \mathrm{IRT1} \quad (6)$$

$$\dot{\mathrm{Fe}}_{\mathrm{ext}}=-\left(k_1\cdot \mathrm{Fe}_{\mathrm{ext}}+k_{19}\cdot \mathrm{Fe}_{\mathrm{ext}}\cdot \mathrm{IRT1}\right)\cdot f_{ext} \quad (7)$$

$$\dot{FIT}=k_{24}\cdot\left(\frac{K_I^{\mathrm{Fe}}}{K_I^{\mathrm{Fe}}+\mathrm{Fe}_{\mathrm{cyt}}}\right)-\left(\frac{V_{max}^{FIT}\cdot FIT}{K_M^{FIT}+FIT}\right) \quad (8)$$

$$\dot{\mathrm{FIT}}=k_{27}\cdot FIT-k_{28}\cdot \mathrm{FIT}-k_{32}\cdot \mathrm{FIT}\cdot \mathrm{TF}+k_{33}\cdot(\mathrm{FIT}\cdot \mathrm{TF}) \quad (9)$$

$$\dot{\mathrm{TF}}=k_{29}-k_{30}\cdot \mathrm{TF}+k_{33}\cdot(\mathrm{FIT}\cdot \mathrm{TF})-k_{32}\cdot \mathrm{FIT}\cdot \mathrm{TF} \quad (10)$$

$$(\mathrm{FIT}\dot{\cdot}\mathrm{TF})=k_{32}\cdot \mathrm{FIT}\cdot \mathrm{TF}-k_{33}\cdot(\mathrm{FIT}\cdot \mathrm{TF}) \quad (11)$$

f_{ext} is the ratio between the cytosolic volume and the external volume.
